# Supplementary material for: Anti-Toxoplasma gondii IgG seroprevalence in the general population in Iran: A systematic review and meta-analysis, 2000–2023
Source: PLoS One. 2024 Aug 29;19(8):e0307941. doi: 10.1371/journal.pone.0307941 (PMC11361691; doi:10.1371/journal.pone.0307941)
Supplement: S1 File — S1 Fig. Funnel plot of standard error by logit event rate to illustrate assessment of publication bias for studies reporting anti-Toxoplasma gondii IgG seroprevalence in the general population of Iran. S2 Fig. Random-effect meta-analysis of pooled estimation of Anti-Toxoplasma gondii IgG seroprevalence in the general population in Iran. S1 Table. Main characteristics of the included studies. (DOC) [file pone.0307941.s001.doc]

**Fig S1.**

**Fig S2.**

**Table S1.**

| **Author** | **Publication year** | **Province** | **Diagnostic Method** | **Sample size** | **IgG positive Cases** | **Prevalence (%)** | **Type of Study** | **Type of population** | **Quality assessment** |
| --- | --- | --- | --- | --- | --- | --- | --- | --- | --- |
| **Shahra. et al [1]** | 2023 | Tehran | ELISA | 200 | 56 | 28 | Case-control | General | Moderate |
| **Rahnama. et al [2]** | 2020 | Fars | ELISA | 130 | 18 | 13.84 | Case-Control | General | Moderate |
| **Sabzevari. et al [3]** | 2017 | Khuzestan | ELISA | 100 | 21 | 21 | Case-Control | General | Moderate |
| **Saki. et al [4]** | 2021 | Khuzestan | ELISA | 54 | 14 | 25.9 | Case-Control | General | Moderate |
| **Mousavi. et al [5]** | 2012 | Sistan and Baluchestan | ELISA | 185 | 19 | 10.27 | Cross-sectional | Pregnant women | Low |
| **Saeedi. et al [6]** | 2007 | Golestan | ELISA | 300 | 145 | 48.33 | Cross-sectional | General | Moderate |
| **Pirali-Kheirabadi. et al [7]** | 2013 | Mazandaran | ELISA | 355 | 315 | 88.73 | Cross-sectional | General | Moderate |
| **Pirali-Kheirabadi. et al [7]** | 2013 | Isfahan | ELISA | 167 | 77 | 46.1 | Cross-sectional | General | Moderate |
| **Pirali-Kheirabadi. et al [7]** | 2013 | Chaharmahal and Bakhtiari | ELISA | 190 | 121 | 63.68 | Cross-sectional | General | Moderate |
| **Rostami. et al [8]** | 2016 | Mazandaran | ELISA | 630 | 465 | 73.8 | Cross-sectional | General | Low |
| **Mahbod. et al [9]** | 2004 | Tehran | ELISA, IFA | 106 | 38 | 35.84 | Cross-sectional | children | Moderate |
| **Kalantari. et al [10]** | 2018 | Mazandaran | ELISA | 138 | 12 | 8.69 | Case-Control | children | Low |
| **Mahami Oskouei. et al [11]** | 2016 | East Azerbaijan | ELISA | 75 | 68 | 90.66 | Case-Control | General | Moderate |
| **Dalimi. et al [12]** | 2012 | East Azerbaijan | ELISA | 300 | 79 | 26.33 | Cross-sectional | Pregnant women | Moderate |
| **Ebrahimzadeh. et al [13]** | 2013 | Sistan and Baluchestan | ELISA | 221 | 68 | 30.76 | Cross-sectional | Pregnant women | Low |
| **Javadi. et al [14]** | 2014 | Qazvin | ELISA, PCR | 195 | 115 | 58.97 | Cross-sectional | Pregnant women | Moderate |
| **Soltani. et al [15]** | 2021 | Khuzestan | ELISA | 88 | 29 | 32.9 | Cross-sectional | Pregnant women | Low |
| **Saki. et al [16]** | 2015 | Khuzestan | ELISA | 130 | 28 | 21.5 | Cross-sectional | Pregnant women | High |
| **Cheraghipour. et al [17]** | 2007 | Lorestan | ELISA | 331 | 95 | 28.7 | Cross-sectional | Pregnant women | Moderate |
| **Akhlaghi. et al [18]** | 2016 | Alborz | Avidity ELISA | 468 | 86 | 18.37 | Cross-sectional | Pregnant women | Moderate |
| **Panah. et al [19]** | 2013 | Mazandaran | ELISA | 1057 | 739 | 69.9 | Cross-sectional | Pregnant women | High |
| **Vakil. et al [20]** | 2013 | Markazi | ELISA | 308 | 117 | 37.98 | Cross-sectional | Pregnant women | High |
| **Shieh. et al [21]** | 2017 | Markazi | ELISA | 261 | 86 | 32.9 | Cross-sectional | Pregnant women | Moderate |
| **Hariri. et al [22]** | 2023 | Ardabil | ELISA | 244 | 54 | 22.1 | Cross-sectional | Pregnant women | Moderate |
| **Kalantari. et al [23]** | 2015 | Mazandaran | ELISA | 175 | 106 | 60.57 | Cross-sectional | Pregnant women | Low |
| **Foulavand. et al [24]** | 2008 | Khuzestan | ELISA | 365 | 138 | 37.8 | Cross-sectional | Pregnant women | Moderate |
| **Manouchehri Naini. et al [25]** | 2006 | Chaharmahal and Bakhtiari | IFA | 384 | 106 | 27.6 | Cross-sectional | Pregnant women | Moderate |
| **Nejad. et al [26]** | 2011 | Lorestan | ELISA | 721 | 250 | 34.67 | Cohort | Pregnant women | Low |
| **Norouzi. et al [27]** | 2017 | Fars | ELISA | 2000 | 172 | 8.6 | Cross-sectional | Pregnant women | Moderate |
| **Portavandazanpur. et al [28]** | 2014 | Kurdistan | ELISA | 146 | 44 | 30.1 | Cross-sectional | Pregnant women | Moderate |
| **Shirdel. et al [29]** | 2017 | Golestan | ELISA | 440 | 155 | 35.2 | Cross-sectional | Pregnant women | Low |
| **Salehi. et al [30]** | 2021 | Razavi Khorasan | ELISA | 252 | 56 | 22.2 | Case-Control | Pregnant women | Low |
| **Dadimoghdam. et al [31]** | 2014 | Golestan | ELISA | 250 | 56 | 22.4 | Cross-sectional | Pregnant women | High |
| **Sharbatkhori. et al [32]** | 2014 | Golestan | ELISA | 555 | 221 | 39.8 | Cross-sectional | Pregnant women | Moderate |
| **Fallah. et al [33]** | 2004 | Hamedan | ELISA | 576 | 193 | 33.5 | Cross-sectional | Pregnant women | Low |
| **Maqsood. et al [34]** | 2013 | Hamedan | ELISA | 350 | 105 | 30 | Cross-sectional | Pregnant women | High |
| **Abdi. et al [35]** | 2008 | Ilam | ELISA | 553 | 247 | 44.66 | Cross-sectional | Pregnant women | High |
| **Maani. et al [36]** | 2017 | Fars | ELISA | 370 | 109 | 29.4 | Cross-sectional | Pregnant women | Low |
| **Akhlaghi. et al [37]** | 2013 | Alborz | ELISA | 400 | 116 | 29 | Cross-sectional | Pregnant women | Moderate |
| **Talari. et al [38]** | 2001 | Isfahan | IFA | 562 | 234 | 41.6 | Cross-sectional | Pregnant women | Moderate |
| **Zalai. et al [39]** | 2014 | Kermanshah | ELISA | 386 | 133 | 34.4 | Cross-sectional | Pregnant women | Moderate |
| **Talari. et al [40]** | 2002 | Isfahan | IFA | 270 | 72 | 26.66 | Cross-sectional | Pregnant women | Moderate |
| **Cheraghipour. et al [41]** | 2010 | Lorestan | ELISA | 390 | 121 | 31 | Cross-sectional | Pregnant women | Moderate |
| **Sharifi. et al [42]** | 2019 | Razavi Khorasan | ELISA | 250 | 58 | 23.2 | Cross-sectional | Pregnant women | Moderate |
| **Hoseini. et al [43]** | 2014 | Mazandaran | ELISA | 289 | 170 | 58.8 | Cross-sectional | Pregnant women | Low |
| **Mohaghegh. et al [44]** | 2016 | North Khorasan | ELISA | 350 | 110 | 31.4 | Cross-sectional | Pregnant women | Moderate |
| **Aliabadi. et al [45]** | 2017 | North Khorasan | ELISA | 1302 | 690 | 53 | Cross-sectional | Pregnant women | Moderate |
| **Eskandarian. et al [46]** | 2017 | Qazvin | IFA | 255 | 160 | 62.7 | Cross-sectional | Pregnant women | High |
| **Maleki. et al [47]** | 2013 | Qom | ELISA, IFA | 200 | 69 | 34.5 | Cross-sectional | Pregnant women | Moderate |
| **Maleki. et al [47]** | 2013 | Qom | ELISA, IFA | 200 | 76 | 38 | Cross-sectional | Pregnant women | Moderate |
| **Haeri. et al [48]** | 2014 | Qom | ELISA | 25 | 20 | 80 | Cross-sectional | Pregnant women | High |
| **Fallahizadeh. et al [49]** | 2018 | Khuzestan | ELISA | 276 | 85 | 30.8 | Cross-sectional | Pregnant women | High |
| **Rahmati-Balaghaleh. et al [50]** | 2019 | Razavi Khorasan | ELISA, Avidity ELISA | 208 | 81 | 38.9 | Cross-sectional | Pregnant women | Moderate |
| **Pashaei Naghadeh. et al [51]** | 2015 | South Khorasan | ELISA | 205 | 80 | 39 | Cross-sectional | Pregnant women | Moderate |
| **Ahmadpour. et al [52]** | 2019 | West Azerbaijan | chemiluminescence immunoassay | 276 | 55 | 19.9 | Cross-sectional | Pregnant women | Moderate |
| **Ghasemloo. et al [53]** | 2014 | Tehran | ELISA | 785 | 135 | 17.2 | Cross-sectional | Pregnant women | Moderate |
| **Gharavi. et al [54]** | 2002 | Tehran | IFA | 4120 | 2804 | 68 | Cross-sectional | Pregnant women | Low |
| **Mozaffari. et al [55]** | 2019 | Tehran | ELISA | 470 | 166 | 35.3 | Cross-sectional | Pregnant women | Low |
| **Hazrati Tape. et al [56]** | 2015 | West Azerbaijan | ELISA | 346 | 98 | 28.3 | Cross-sectional | Pregnant women | Moderate |
| **Khameneh. et al [57]** | 2016 | West Azerbaijan | ELISA | 156 | 44 | 28.2 | Cross-sectional | Pregnant women | High |
| **Mohammadnejad. et al [58]** | 2018 | West Azerbaijan | ELISA | 620 | 114 | 18.4 | Cross-sectional | Pregnant women | Moderate |
| **Raissi. et al [59]** | 2020 | Ilam | ELISA | 189 | 75 | 39.7 | Cross-sectional | Pregnant women | Moderate |
| **Anvari. et al [60]** | 2013 | Yazd | ELISA | 181 | 58 | 32 | Cross-sectional | Pregnant women | High |
| **Sharifi mod. et al [61]** | 2016 | Sistan and Baluchestan | IFA | 200 | 54 | 27 | Cross-sectional | Pregnant women | Moderate |
| **Ebrahimzadeh. et al [62]** | 2011 | Sistan and Baluchestan | ELISA | 221 | 68 | 30.8 | Cross-sectional | Pregnant women | Moderate |
| **Sharifi -Mood. et al [63]** | 2004 | Sistan and Baluchestan | IFA | 200 | 54 | 27 | Cross-sectional | Pregnant women | Moderate |
| **Sotoudeh Jahormi. et al [64]** | 2002 | Hormozgān | ELISA | 418 | 133 | 31.8 | Cross-sectional | Pregnant women | High |
| **Firouz. et al [65]** | 2014 | Mazandaran | ELISA, CLIA | 50 | 26 | 52 | Cross-sectional | Pregnant women | Low |
| **Babaie. et al [66]** | 2013 | Razavi Khorasan | ELISA | 419 | 144 | 34.7 | Cross-sectional | Pregnant women | High |
| **Zavari. et al [67]** | 2015 | Kerman | ELISA | 240 | 73 | 30.4 | Cross-sectional | Pregnant women | Moderate |
| **Sadeghi Dehkordi. et al [68]** | 2022 | Kurdistan | ELISA | 146 | 44 | 30.1 | Cross-sectional | Pregnant women | High |
| **Ahmadpour. et al [69]** | 2017 | Lorestan | ELISA | 264 | 66 | 25 | Cross-sectional | Pregnant women | Moderate |
| **Hajsoleimani. et al [70]** | 2012 | Zanjan | ELISA | 500 | 186 | 37.2 | Cross-sectional | Pregnant women | Moderate |
| **Akhlaghi. et al [71]** | 2013 | Qom | ELISA, IFA | 200 | 76 | 38 | Cross-sectional | Pregnant women | Low |
| **Naini. et al [72]** | 2018 | Chaharmahal and Bakhtiari | ELISA | 384 | 106 | 27.6 | Cross-sectional | Pregnant women | Moderate |
| **Jahantigh. et al [73]** | 2020 | Sistan and Baluchestan | ELISA | 90 | 13 | 14.4 | Cross-sectional | Pregnant women | Moderate |
| **Noorbakhsh. et al [74]** | 2002 | Tehran | ELISA | 140 | 48 | 34.3 | Cross-sectional | Pregnant women | High |
| **Khademi. et al [75]** | 2019 | Hormozgan | ELISA | 360 | 100 | 27.8 | Cross-sectional | Pregnant women | High |
| **Shiadeh. et al [76]** | 2016 | Tehran | ELISA | 244 | 125 | 51.2 | Case-Control | Pregnant women | Moderate |
| **Rostaminejad. et al [77]** | 2013 | Tehran | ELISA | 496 | 154 | 31 | Case-Control | Pregnant women | High |
| **Abdollahian. et al [78]** | 2017 | Razavi Khorasan | ELISA | 350 | 120 | 34.3 | Case-control | Pregnant women | Low |
| **Soleymani. et al [79]** | 2021 | Mazandaran | ELISA | 350 | 296 | 84.6 | Cross-sectional | Pregnant women | Moderate |
| **Saraei. et al [80]** | 2009 | Qazvin | IFA | 400 | 136 | 34 | Cross-sectional | Pregnant women | High |
| **Dehgani. et al [81]** | 2016 | Mazandaran | IFA | 600 | 190 | 31.7 | Cross-sectional | General | Moderate |
| **Rasti. et al [82]** | 2016 | Qom | ELISA | 120 | 21 | 17.5 | Case-Control | General | Moderate |
| **Soltani. et al [83]** | 2013 | Khuzestan | ELISA | 100 | 26 | 26 | Case-Control | General | Moderate |
| **Kawakb. et al [84]** | 2009 | Tehran | ELISA | 31 | 19 | 61.3 | Case-Control | General | Low |
| **Zarean. et al [85]** | 2022 | Razavi Khorasan | ELISA | 40 | 4 | 10 | Case-Control | General | Moderate |
| **Mojadadi. et al [86]** | 2016 | Razavi Khorasan | ELISA | 137 | 18 | 13.1 | Cross-sectional | General | Moderate |
| **Mohammadi. et al [87]** | 2015 | Kurdistan | ELISA | 600 | 169 | 28.2 | Cross-sectional | General | Moderate |
| **Sharif. et al [88]** | 2010 | Mazandaran | ELISA | 1209 | 266 | 22 | Case-Control | General | High |
| **Alipour. et al [89]** | 2011 | Tehran | ELISA | 62 | 23 | 37 | Case-Control | General | Moderate |
| **Banihashem. et al [90]** | 2023 | Tehran | ELISA | 75 | 38 | 50.7 | Cross-sectional | General | Moderate |
| **Khademvatan. et al [91]** | 2014 | Khuzestan | ELISA | 95 | 45 | 47.4 | Case-Control | children | Low |
| **Ebadi. et al [92]** | 2014 | Tehran | ELISA | 152 | 63 | 41.4 | Case-Control | General | High |
| **Ansari‐Lari. et al [93]** | 2017 | Fars | ELISA | 152 | 41 | 27 | Case-Control | General | High |
| **Meftahi. et al [94]** | 2021 | Razavi Khorasan | ELISA | 40 | 18 | 45 | Case-Control | General | High |
| **Daryani. et al [95]** | 2010 | Mazandaran | ELISA | 99 | 25 | 25.2 | Case-Control | General | Moderate |
| **Babaie. et al [96]** | 2017 | Tehran | ELISA | 63 | 24 | 38.1 | Case-Control | blood donors | High |
| **Taghizadeh. et al [97]** | 2017 | Fars | ELISA | 503 | 37 | 7.3 | Case-Control | General | Moderate |
| **Makiani. et al [98]** | 2012 | Hormozgān | ELISA | 600 | 252 | 42 | Cross-sectional | General | High |
| **Rajaii. et al [99]** | 2013 | East Azerbaijan | IFA | 211 | 178 | 84.4 | Cross-sectional | General | High |
| **Rajaii. et al [99]** | 2013 | East Azerbaijan | IFA | 162 | 110 | 67.9 | Cross-sectional | General | Low |
| **Rajaii. et al [99]** | 2013 | East Azerbaijan | IFA | 86 | 71 | 82.5 | Cross-sectional | Pregnant women | Moderate |
| **Rajaii. et al [99]** | 2013 | East Azerbaijan | IFA | 105 | 59 | 56.2 | Cross-sectional | Pregnant women | Moderate |
| **Rajaii. et al [99]** | 2013 | East Azerbaijan | IFA | 110 | 45 | 40.9 | Cross-sectional | Pregnant women | Moderate |
| **Rajaii. et al [99]** | 2013 | East Azerbaijan | IFA | 985 | 435 | 44.2 | Cross-sectional | Pregnant women | Moderate |
| **Ramezani. et al [100]** | 2016 | Tehran | ELISA | 40 | 26 | 65 | Cross-sectional | Pregnant women | Moderate |
| **Arbabi. et al [101]** | 2002 | Isfahan | IFA | 340 | 207 | 60.9 | Cross-sectional | Pregnant women | Moderate |
| **Arbabi. et al [101]** | 2002 | Isfahan | IFA | 300 | 140 | 46.7 | Cross-sectional | Pregnant women | Moderate |
| **Turkan. et al [102]** | 2008 | Isfahan | IFA | 120 | 35 | 29.2 | Cross-sectional | General | Moderate |
| **Manochehri Nayini. et al [103]** | 2011 | Chaharmahal and Bakhtiari | ELISA | 427 | 202 | 47.3 | Cross-sectional | Pregnant women | Moderate |
| **Manochehri Nayini. et al [103]** | 2011 | Chaharmahal and Bakhtiari | ELISA | 119 | 29 | 24.7 | Cross-sectional | General | Moderate |
| **Manochehri Nayini. et al [103]** | 2011 | Chaharmahal and Bakhtiari | ELISA | 108 | 45 | 41.7 | Cross-sectional | General | High |
| **Manochehri Nayini. et al [103]** | 2011 | Chaharmahal and Bakhtiari | ELISA | 82 | 23 | 28 | Cross-sectional | General | Moderate |
| **Manochehri Nayini. et al [103]** | 2011 | Chaharmahal and Bakhtiari | ELISA | 57 | 11 | 19.3 | Cross-sectional | General | Moderate |
| **Manochehri Nayini. et al [103]** | 2011 | Chaharmahal and Bakhtiari | ELISA | 197 | 29 | 14.7 | Cross-sectional | General | Moderate |
| **Shaddel. et al [104]** | 2014 | Fars | ELISA | 250 | 58 | 23.2 | Cross-sectional | General | Moderate |
| **Razavi. et al [105]** | 2003 | Fars | IFA | 87 | 24 | 27.6 | Cross-sectional | General | Moderate |
| **Dorri. et al [106]** | 2017 | Sistan and Baluchestan | ELISA | 76 | 33 | 43.4 | Case-Control | General | Moderate |
| **Youssefi. et al [107]** | 2018 | Razavi Khorasan | ELISA | 91 | 53 | 58.2 | Cross-sectional | blood donors | High |
| **Salahi-Moghaddam. et al [108]** | 2009 | Tehran | IFA | 1187 | 812 | 68.4 | Cross-sectional | General | Moderate |
| **Soltani. et al [109]** | 2018 | Khuzestan | ELISA | 496 | 188 | 37.9 | Cross-sectional | General | High |
| **NEMATOLLAHI. et al [110]** | 2022 | Yazd | ELISA | 300 | 32 | 10.7 | Cross-sectional | General | High |
| **Kheirandish. et al [111]** | 2019 | Lorestan | ELISA | 240 | 111 | 46.2 | Case-Control | General | Moderate |
| **Sadaghian. et al [112]** | 2016 | East Azerbaijan | ELISA | 80 | 27 | 33.7 | Case-Control | General | Low |
| **Sadaghian. et al [112]** | 2017 | East Azerbaijan | ELISA | 80 | 29 | 36.2 | Case-Control | Pregnant women | Moderate |
| **Saki. et al [113]** | 2020 | Khuzestan | ELISA | 100 | 21 | 21 | Case-Control | Pregnant women | High |
| **Jafari. et al [114]** | 2012 | East Azerbaijan | ELISA | 171 | 60 | 35.1 | Cross-sectional | children | High |
| **Fatollahzadeh. et al [115]** | 2016 | East Azerbaijan | ELISA | 5770 | 1871 | 32.4 | Cross-sectional | General | High |
| **Abdul Nasser. et al [116]** | 2005 | East Azerbaijan | IFA | 146 | 61 | 41.8 | Cross-sectional | General | Moderate |
| **Gadmagahi. et al [117]** | 2013 | Tehran | ELISA | 300 | 85 | 28.3 | Cross-sectional | General | Moderate |
| **Shariat. et al [118]** | 2019 | Tehran | ELISA | 980 | 120 | 12.2 | Cross-sectional | General | Low |
| **Moghimi. et al [119]** | 2015 | Yazd | ELISA | 144 | 4 | 2.8 | Cross-sectional | General | High |
| **Yousefi. et al [120]** | 2017 | Khuzestan | ELISA | 205 | 41 | 20 | Case-Control | General | High |
| **Hanifehpour. et al [121]** | 2019 | Chaharmahal and Bakhtiari | ELISA | 235 | 82 | 34.9 | Case-Control | Pregnant women | Moderate |
| **Soltani. et al [122]** | 2021 | Khuzestan | ELISA | 50 | 17 | 34 | Case-Control | General | High |
| **Soltani. et al [122]** | 2021 | Khuzestan | ELISA | 100 | 29 | 29 | Case-Control | General | Low |
| **Mohammadpour. et al [123]** | 2018 | Alborz | ELISA | 343 | 110 | 32.1 | Cross-sectional | General | High |
| **Rasti. et al [124]** | 2016 | Tehran | ELISA | 98 | 28 | 28.6 | Case-Control | General | Low |
| **Arefkhah. et al [125]** | 2020 | Kohgiluyeh and Boyer-Ahmad | ELISA | 1005 | 174 | 17.3 | Cross-sectional | General | Low |
| **Ghasemi. et al [126]** | 2016 | Tehran | ELISA | 110 | 29 | 26.4 | Case-Control | General | Moderate |
| **Soltani. et al [127]** | 2020 | Khuzestan | ELISA | 100 | 29 | 29 | Case-Control | Pregnant women | Moderate |
| **Kalantari. et al [128]** | 2020 | Fars | ELISA | 504 | 36 | 7.1 | Cross-sectional | General | Moderate |
| **Farhang. et al [129]** | 2014 | East Azerbaijan | ELISA | 100 | 34 | 34 | Cross-sectional | Pregnant women | Moderate |
| **Rasouli. et al [130]** | 2010 | West Azerbaijan | CLIA | 200 | 94 | 47 | Cross-sectional | General | Moderate |
| **Sadaghian. et al [131]** | 2016 | West Azerbaijan | ELISA | 195 | 88 | 45.1 | Cross-sectional | General | High |
| **Tappeh. et al [132]** | 2017 | West Azerbaijan | ELISA | 270 | 102 | 37.8 | Cross-sectional | General | Moderate |
| **Fakhrieh Kashan. et al [133]** | 2019 | Isfahan | ELISA | 70 | 16 | 22.8 | Cross-sectional | General | Moderate |
| **Mousavi-Hasanzadeh. et al[134]** | 2020 | Markazi | ELISA | 599 | 198 | 33 | Cross-sectional | General | Moderate |
| **Mousavi-Hasanzadeh. et al [134]** | 2020 | Markazi | ELISA | 49 | 24 | 49 | Cross-sectional | Blood Donors | Moderate |
| **Mousavi-Hasanzadeh. et al [134]** | 2020 | Markazi | ELISA | 48 | 6 | 12.5 | Cross-sectional | General | High |
| **Mousavi-Hasanzadeh. et al [134]** | 2020 | Markazi | ELISA | 48 | 25 | 52.1 | Cross-sectional | General | Moderate |
| **Mousavi-Hasanzadeh. et al [134]** | 2020 | Markazi | ELISA | 32 | 15 | 46.9 | Cross-sectional | General | Moderate |
| **Mousavi-Hasanzadeh. et al [134]** | 2020 | Markazi | ELISA | 48 | 28 | 58.3 | Cross-sectional | General | Moderate |
| **Rahimkhani. et al [135]** | 2021 | Tehran | ELISA | 103 | 19 | 18.4 | Cross-sectional | General | Moderate |
| **Saidi. et al [136]** | 2002 | Golestan | ELISA | 300 | 145 | 48.3 | Cross-sectional | General | Moderate |
| **Rezaei. et al [137]** | 2017 | Semnan | ELISA | 400 | 50 | 12.5 | Cross-sectional | General | Moderate |
| **Bokharaei-Salim. et al [138]** | 2023 | Tehran | ELISA | 278 | 69 | 24.8 | Case-Control | General | Moderate |
| **Bokharaei-Salim. et al [138]** | 2023 | Tehran | ELISA | 182 | 22 | 12.1 | Case-Control | Pregnant women | Moderate |
| **Ghorbani. et al [139]** | 2013 | Kohgiluyeh and Boyer-Ahmad | ELISA | 410 | 36 | 8.8 | Cross-sectional | Pregnant women | Moderate |
| **Bafghi. et al [140]** | 2015 | Yazd | ELISA | 712 | 171 | 24 | Cross-sectional | children | Low |
| **Bafghi. et al [141]** | 2020 | Yazd | ELISA | 200 | 37 | 18.5 | Cross-sectional | children | Low |
| **Hajipour. et al [142]** | 2023 | Yazd | ELISA | 185 | 35 | 18.9 | Cross-sectional | General | Moderate |
| **Gharavi. et al [143]** | 2018 |  | ELISA | 882 | 497 | 56.3 | Cross-sectional | General | Moderate |
| **Haghighi. et al [144]** | 2020 | Sistan and Baluchestan | ELISA | 96 | 26 | 27.1 | Case-Control | General | Moderate |
| **Khabisi. et al [145]** | 2022 | Sistan and Baluchestan | ELISA | 1074 | 250 | 23.3 | Cross-sectional | Pregnant women | High |
| **Jafari-Modrek. et al [146]** | 2019 | Sistan and Baluchestan | ELISA | 80 | 8 | 10 | Cross-sectional | children | Moderate |
| **Sardarian. et al [147]** | 2019 | Hamadan | ELISA | 653 | 167 | 25.8 | Cross-sectional | General | Moderate |
| **Soltni. et al [148]** | 2022 | Khuzestan | ELISA | 252 | 100 | 39.7 | Cross-sectional | General | Moderate |
| **Maraghi. et al [149]** | 2013 | Khuzestan | ELISA | 240 | 28 | 11.7 | Cross-sectional | children | Moderate |
| **Jahromi. et al [150]** | 2007 | Hormozgan | ELISA | 251 | 83 | 33.1 | Case-control | Pregnant women | Moderate |
| **Saki. et al [151]** | 2021 | Khuzestan | ELISA | 200 | 43 | 21.5 | Cross-sectional | General | Low |
| **Aali. et al [152]** | 2010 | Kerman | ELISA | 112 | 27 | 24.1 | Case-control | General | Low |
| **Ebadi. et al [153]** | 2011 | Fars | ELISA | 50 | 7 | 14 | Case-control | General | Low |
| **Raissi. et al [59]** | 2020 | Ilam | ELISA | 189 | 19 | 10 | Cross-sectional | General | Moderate |
| **Ziaee. et al [154]** | 2008 | Khuzestan | ELISA | 400 | 103 | 25.7 | Cross-sectional | General | Low |
| **Afroogh. et al [155]** | 2007 | Khuzestan | ELISA | 390 | 120 | 30.8 | Cross-sectional | General | Low |
| **Sohrabi. et al [156]** | 2007 | Khuzestan | ELISA | 79 | 28 | 35.4 | Cross-sectional | General | Moderate |
| **Rafiee. et al[157]** | 2005 | Khuzestan | ELISA | 259 | 12 | 4.6 | Cross-sectional | General | Moderate |
| **Fallah. et al [158]** | 2014 | East Azarbaijan | ELISA | 549 | 88 | 16 | Cross-sectional | General | Moderate |
| **Shahighi. et al [159]** | 2021 | Alborz | ELISA | 400 | 81 | 20.2 | Cross-sectional | General | Low |
| **Mahami-Oskouei. et al [160]** | 2016 | East Azarbaijan | ELISA | 75 | 47 | 62.7 | Case-control | General | Moderate |
| **Menati Rashno. et al [161]** | 2016 | Lorestan | ELISA | 87 | 49 | 56.3 | Case-control | Pregnant women | Moderate |
| **Mohammadi. et al[162]** | 2015 | Markazi | ELISA | 400 | 97 | 24.2 | Cross-sectional | General | Moderate |
| **Daryani. et al [163]** | 2004 | Ardabil | IFA | 504 | 175 | 34.7 | Cross-sectional | General | Low |
| **Alimohammadi. et al [164]** | 2008 | Ardabil | ELISA | 227 | 115 | 50.7 | Cross-sectional | General | Low |
| **Afsharpeyman. et al [165]** | 2014 | Tehran | ELISA | 40 | 1 | 2.5 | Case-control | General | Low |
| **Azizi. et al [166]** | 2020 | Khuzestan | ELISA | 50 | 4 | 8 | Case-control | General | Low |
| **Hamid. et al [167]** | 2020 | Khuzestan | ELISA | 50 | 5 | 10 | Case-control | General | Low |
| **Kalantari. et al [168]** | 2017 | Mazandaran |  | 322 | 90 | 27.9 | Cross-sectional | General | Moderate |
| **Kanani. et al [169]** | 2022 | South Khorasan | ELISA | 300 | 25 | 8.3 | Cross-sectional | General | Low |
| **Rezadadeh. et al [170]** | 2015 | South Khorasan | ELISA | 519 | 101 | 19.5 | Cross-sectional | children | Low |
| **Davami. et al [171]** | 2015 | Fars | ELISA | 400 | 54 | 13.5 | Cross-sectional | children | Low |
| **Sarkari. et al [172]** | 2013 | Fars | ELISA | 1480 | 182 | 12.3 | Cross-sectional | children | Low |
| **Asfaram. et al [173]** | 2021 | Ardabil | ELISA | 462 | 150 | 32.5 | Cross-sectional | General | Low |
| **Moshfe. et al [174]** | 2018 | kohgiluyeh and boyer ahmad | ELISA | 285 | 46 | 16.1 | Cross-sectional | General | Low |
| **Barazesh. et al [175]** | 2023 | Bushehr | ELISA | 243 | 76 | 31.3 | Cross-sectional | General | Low |
| **Manouchehri Naeini. et al [176]** | 2019 | Chaharmahal and Bakhtiari | ELISA | 385 | 146 | 37.9 | Cross-sectional | blood donor | Low |
| **Ferdowsi. et al [177]** | 2013 | Khorasan Razavi | ELISA | 300 | 48 | 16 | Cross-sectional | blood donor | Low |
| **Gholami. et al [178]** | 2015 | Hamedan | ELISA | 540 | 294 | 54.4 | Cross-sectional | blood donor | Low |
| **Sadooghian. et al [179]** | 2017 | Khorasan Razavi | ELISA | 491 | 184 | 37.5 | Cross-sectional | blood donor | Low |
| **Zainodini. et al [180]** | 2014 | Kerman | ELISA | 235 | 80 | 34 | Cross-sectional | blood donor | Low |
| **Mahmoudvand. et al [181]** | 2015 | Kerman | ELISA | 500 | 144 | 28.8 | Cross-sectional | blood donor | Low |
| **Saki. et al [182]** | 2019 | Khuzestan | ELISA | 380 | 131 | 34.5 | Cross-sectional | blood donor | Low |
| **Bahhaj. et al [183]** | 2017 | East Azarbaijan | CLIA | 194 | 75 | 38.6 | Cross-sectional | blood donor | Low |
| **Ormazdi. et al [184]** | 2010 | Tehran | ELISA | 250 | 132 | 52.8 | Cross-sectional | blood donor | Low |
| **Shaddel. et al [185]** | 2014 | Tehran | ELISA | 223 | 86 | 38.6 | Cross-sectional | blood donor | Moderate |
| **Kalantari. et al [186]** | 2018 | Mazandaran | ELISA | 500 | 316 | 63.2 | Cross-sectional | blood donor | Low |
| **Jafari Modrek. et al [187]** | 2014 | Sistan va baluchestan | ELISA | 375 | 94 | 25.1 | Cross-sectional | blood donor | Low |
| **Zarean. et al [188]** | 2017 | Khorasan Razavi | ELISA | 500 | 125 | 25 | Cross-sectional | blood donor | Low |
| **Ali-asghari. et al [189]** | 2013 | North Khorasan | ELISA | 215 | 44 | 20.5 | Cross-sectional | blood donor | Moderate |
| **Shafiei. et al [190]** | 2022 | North Khorasan | ELISA | 361 | 22 | 6.1 | Cross-sectional | blood donor | Moderate |
| **Asgari. et al [191]** | 2023 |  | ELISA | 124 | 12 | 9.7 | Case-control | blood donor | Low |
| **Arefkhah. et al [192]** | 2022 | Fars | ELISA | 124 | 15 | 12.1 | Case-control | blood donor | Moderate |
| **Kalantari. et al [193]** | 2015 | Mazandaran | ELISA | 66 | 47 | 71.2 | Cross-sectional | blood donor | Low |
| **Nikbaksh. et al [194]** | 2022 | Mazandaran | ELISA | 91 | 87 | 95.6 | Cross-sectional | General | Low |
| **Mardani. et al [195]** | 2015 | Khuzestan | ELISA | 110 | 47 | 42.7 | Cross-sectional | children | Low |
| **Fouladvand. et al [196]** | 2010 | Bushehr | ELISA | 516 | 114 | 22.1 | Cross-sectional | General | Moderate |
| **Fouladvand. et al [197]** | 2010 | Bushehr | ELISA | 491 | 54 | 10.1 | Cross-sectional | General | Low |
| **Fouladvand. et al [196]** | 2010 | Bushehr | ELISA | 303 | 71 | 23.4 | Cross-sectional | General | Low |
| **Beheshtipour. et al [198]** | 2019 | Kurdistan | ELISA | 53 | 6 | 11.3 | Cross-sectional | General | Low |
| **Mahmoudi. et al [199]** | 2019 | Guilan | ELISA | 150 | 96 | 64 | Case-control | General | Moderate |
| **Ghasemian. et al [200]** | 2007 | Khuzestan | ELISA | 252 | 81 | 32.1 | Cross-sectional | General | Low |
| **Getso. et al [201]** | 2015 | Khuzestan | ELISA | 600 | 67 | 11.2 | Cross-sectional | General | Low |
| **Khademvatan. et al [202]** | 2020 | West Azerbaijan | ELISA | 336 | 127 | 37.8 | Cross-sectional | General | Low |
| **Manouchehri-Naeini. et al [103]** | 2011 | Chaharmahal and Bakhtiari | ELISA | 990 | 339 | 34.2 | Cross-sectional | General | Low |
| **Manouchehri Naeini. et al [203]** | 2013 | Chaharmahal and Bakhtiari | ELISA | 338 | 126 | 37.3 | Cross-sectional | General | Low |
| **Sobati. et al [204]** | 2020 | Qom | CLIA | 202 | 133 | 65.8 | Cross-sectional | General | Low |
| **Raissi. et al [205]** | 2020 | Sistan va baluchestan | ELISA | 869 | 141 | 16.2 | Cross-sectional | General | Low |
| **Rezavand. et al [206]** | 2016 | Ilam | ELISA | 90 | 34 | 37.8 | Cross-sectional | General | Low |
| **Mardani. et al [207]** | 2013 | Qom | ELISA | 600 | 257 | 42.8 | Cross-sectional | General | Low |
| **Mardani. et al [207]** | 2013 | Qom | IFA | 600 | 246 | 41 | Cross-sectional | General | Low |
| **Fasihi-Karami. et al [208]** | 2023 |  | ELISA | 100 | 17 | 17 | Case-control | General | Low |
| **Afsharpaiman. et al [209]** | 2016 | Tehran | ELISA | 48 | 1 | 2.1 | Cross-sectional | children | Low |
| **Khademvatan. et al [210]** | 2018 | Khuzestan | ELISA | 83 | 20 | 24.1 | Cross-sectional | General | Moderate |
| **Khodashenas. et al [211]** | 2019 | Markazi and Hamadan | ELISA | 99 | 39 | 39.4 | Case-control | Pregnant women | Moderate |
| **Nasirpour. et al [212]** | 2020 | Lorestan | ELISA | 87 | 49 | 56.3 | Case-control | Pregnant women | Moderate |
| **Shamsinia. et al [213]** | 2019 | Tehran | ELISA | 94 | 16 | 17 | Cross-sectional | General | Moderate |
| **KHALILI. et al [214]** | 2018 | East Azarbaijan | ELISA | 100 | 59 | 59 | Case-control | children | Moderate |
| **Shirbazou. et al [215]** | 2013 |  | ELISA | 93 | 38 | 40.9 | Case-control | children | Moderate |
| **Saki. et al [216]** | 2016 | Khuzestan | ELISA | 110 | 24 | 21.8 | Case-control | General | Low |
| **Saki. et al [216]** | 2016 | Khuzestan | IFA | 110 | 21 | 19.1 | Case-control | General | Low |
| **Bahmani. et al [217]** | 2020 | Kurdistan | ELISA | 90 | 14 | 15.5 | Case-control | Pregnant women | Moderate |
| **Khademvatan. et al [218]** | 2012 | Khuzestan | ELISA | 237 | 112 | 47.2 | Cross-sectional | General | Low |
| **Saki. et al [219]** | 2013 | Khuzestan | ELISA | 100 | 26 | 26 | Cross-sectional | General | Moderate |
| **Mansouri. et al [220]** | 2003 | Kermanshah | ELISA | 1837 | 668 | 36.4 | Cross-sectional | General | Low |
| **Alizadeh Khatir. et al [221]** | 2021 | Mazandaran | ELISA | 88 | 42 | 47.7 | Case-control | Pregnant women | Low |
| **Allahdin. et al [222]** | 2015 | Khuzestan | ELISA | 144 | 39 | 27.1 | Cross-sectional | Pregnant women | Low |
| **Arefkhah. et al [223]** | 2019 | Fars | ELISA | 617 | 23 | 3.7 | Cross-sectional | Pregnant women | Low |
| **Hatam. et al [224]** | 2005 | Fars | ELISA | 947 | 96 | 10.1 | Cross-sectional | General | Moderate |
| **Rahimi‑Esboei. et al [225]** | 2021 | Mazandaran | ELISA | 500 | 133 | 26.6 | Cross-sectional | General | Moderate |
| **Sharifi. et al [226]** | 2018 | Khorasan Razavi | ELISA | 306 | 104 | 34 | Cross-sectional | General | Low |
| **Mohammadi sardeh. et al [227]** | 2014 | Guilan | ELISA | 800 | 261 | 32.6 | Cross-sectional | General | Low |
| **Heydari. et al [228]** | 2011 | Khorasan Razavi | ELISA | 240 | 35 | 14.6 | Cross-sectional | General | Low |
| **Rezayi Cherati. et al [229]** | 2021 | Golestan | ELISA | 551 | 306 | 55.5 | Cross-sectional | children | Low |
| **Rabiee. et al [230]** | 2003 | Hamadan | IFA | 360 | 140 | 38.9 | Cross-sectional | General | Moderate |
| **Hamidi. et al [231]** | 2015 | Hamadan | ELISA | 2523 | 681 | 27 | Cross-sectional | General | Low |
| **Noorbakhsh. et al [232]** | 2008 | Tehran | ELISA | 63 | 30 | 47.6 | Case-control | General | Low |
| **Hamidi. et al [233]** | 2015 | East Azarbaijan | ELISA | 50 | 34 | 68 | Cross-sectional | General | Low |
| **Kazemi. et al [234]** | 2018 | Khuzestan | ELISA | 80 | 22 | 27.5 | Cross-sectional | General | Low |
| **Kazemi. et al [234]** | 2018 | Khuzestan | CLIA | 80 | 13 | 16.2 | Cross-sectional | General | Low |
| **Soltani. et al [235]** | 2020 | Khuzestan | ELISA | 100 | 23 | 23 | Case-control | General | Moderate |
| **Seyyedpour. et al [236]** | 2016 | Mazandaran | ELISA | 145 | 132 | 91 | Cross-sectional | General | Low |
| **Mirahmadi. et al [237]** | 2021 | Sistan va baluchestan | ELISA | 119 | 28 | 23.5 | Case-control | General | Moderate |
| **Ebrahim Zadeh. et al [238]** | 2014 | Sistan va baluchestan | ELISA | 37 | 11 | 29.7 | Case-control | Pregnant women | Low |
| **Bayani. et al [239]** | 2012 | Mazandaran | ELISA | 50 | 38 | 76 | Cross-sectional | children | Low |
| **Saadat. et al [240]** | 2020 | Guilan | ELISA | 150 | 97 | 64.7 | Case-control | General | Low |
| **Fallahizadeh. et al [241]** | 2018 | Khuzestan | ELISA | 82 | 25 | 30.5 | Cross-sectional | General | Low |
| **Alavi. et al [242]** | 2013 | Khuzestan | ELISA | 84 | 34 | 40.5 | Case-control | General | Low |
| **Abdollahi. et al [243]** | 2013 | Tehran | ELISA | 100 | 62 | 62 | Case-control | General | Low |
| **Daryani. et al [244]** | 2011 | Mazandaran | ELISA | 78 | 59 | 75.6 | Cross-sectional | General | Low |
| **Nikbakht. et al [245]** | 2022 | kohgiluyeh and boyer ahmad | ELISA | 64 | 10 | 15.6 | Case-control | General | Low |
| **Yazdani. et al [246]** | 2018 | Isfahan | ELISA | 85 | 29 | 34.1 | Case-control | General | Low |
| **Azami. et al [247]** | 2019 | Ilam | ELISA | 797 | 186 | 23.3 | Cross-sectional | General | Low |
| **Kamran. et al [248]** | 2014 | Ilam | IFA | 260 | 55 | 21.1 | Cross-sectional | General | Low |
| **Eskandarian. et al [249]** | 2017 | Isfahan | ELISA | 80 | 27 | 33.7 | Case-control | General | Low |
| **Mostafavi. et al [250]** | 2011 | Isfahan | ELISA | 599 | 248 | 41.4 | Cross-sectional | General | Low |
| **Mohaghegh. et al [251]** | 2015 | Isfahan | ELISA | 716 | 288 | 40.2 | Cross-sectional | General | Moderate |
| **Jafari. et al [252]** | 2017 | Isfahan | CLIA | 1243 | 325 | 26.1 | Cross-sectional | General | Moderate |
| **Mostafavi. et al [253]** | 2012 | Isfahan | ELISA | 217 | 103 | 47.5 | Cross-sectional | General | Low |
| **Mahmoodi. et al [254]** | 2003 | Isfahan | IFA | 414 | 76 | 18.3 | Cross-sectional | General | Low |
| **Davami. et al [255]** | 2014 | Fars | ELISA | 403 | 52 | 12.9 | Cross-sectional | General | Low |
| **Fallah. et al [256]** | 2005 | East Azarbaijan | IFA | 1000 | 218 | 21.8 | Cross-sectional | General | Low |
| **Barghamadi. et al [257]** | 2022 | Khorasan Razavi | ELISA | 85 | 16 | 18.8 | Cross-sectional | General | Low |
| **Parvizpour. et al [258]** | 2010 | Kurdistan | ELISA | 201 | 54 | 26.9 | Cross-sectional | General | Low |
| **Arbabi. et al [259]** | 2009 | Isfahan | IFA | 400 | 82 | 20.5 | Cross-sectional | General | Low |
| **Hooshyar. et al [260]** | 2014 | Isfahan | ELISA | 434 | 123 | 28.3 | Cross-sectional | General | Low |
| **Rasti. et al [261]** | 2015 | Isfahan | ELISA | 798 | 341 | 42.7 | Cohort | General | Low |
| **Keshavarz. et al [262]** | 2000 | Kerman | IFA | 690 | 324 | 46.9 | Cross-sectional | General | Moderate |
| **Hajghani. et al [263]** | 2008 | Kerman | ELISA | 549 | 93 | 16.9 | Cross-sectional | General | Moderate |
| **Narooi Dehnavi. et al [264]** | 2018 | Kerman | ELISA | 186 | 20 | 10.7 | Cross-sectional | General | Low |
| **Narooi Dehnavi. et al [264]** | 2018 | Kerman | ELISA | 186 | 10 | 5.4 | Cross-sectional | General | Low |
| **Tavakoli Kareshk. et al [265]** | 2016 | Kerman | ELISA | 300 | 31 | 10.3 | Cross-sectional | General | Low |
| **Pouyanmehr. et al [266]** | 2018 | Kermanshah | ELISA | 315 | 40 | 12.7 | Cross-sectional | Pregnant women | Low |
| **Pouyanmehr. et al [267]** | 2020 | Kermanshah | ELISA | 2470 | 327 | 13.2 | Cross-sectional | General | Low |
| **Kalani. et al [268]** | 2021 | Kermanshah | ELISA | 1228 | 294 | 23.9 | Cross-sectional | General | Low |
| **Kalani. et al [268]** | 2021 | Kermanshah | ELISA | 170 | 41 | 24.1 | Cross-sectional | Pregnant women | Moderate |
| **Almasian. et al [269]** | 2014 | Lorestan | IFA | 1000 | 831 | 83.1 | Cross-sectional | General | Low |
| **Badparva. et al [270]** |  | Lorestan | IFA | 452 | 200 | 44.2 | Cross-sectional | General | Low |
| **Yad Yad. et al [271]** | 2014 | Khuzestan | ELISA | 501 | 137 | 27.3 | Cross-sectional | Pregnant women | Moderate |
| **Mamishi. et al [272]** | 2003 | Tehran | IFA | 446 | 25 | 5.6 | Cross-sectional | General | Moderate |
| **Mehrabani. et al [273]** | 2014 | Fars | IFA | 357 | 44 | 12.3 | Cross-sectional | General | Low |
| **Khayat Nouri. et al [274]** | 2009 | East Azarbaijan | ELISA | 150 | 17 | 11.3 | Case-control | General | Moderate |
| **Nasimi. et al [275]** | 2021 | Khorasan Razavi | ELISA | 417 | 80 | 19.2 | Cross-sectional | General | Moderate |
| **Hosseini. et al [276]** | 2020 | Mazandaran | ELISA | 400 | 294 | 73.5 | Cross-sectional | General | Low |
| **Sharif. et al [277]** | 2016 | Mazandaran | ELISA | 1832 | 1018 | 55.7 | Cross-sectional | Pregnant women | Low |
| **Khalili. et al [278]** | 2014 | Chahrmahal Va Bakhtiari | ELISA | 50 | 14 | 28 | Case-control | General | Low |
| **Soltan mohammadzadeh. et al [279]** | 2002 | Ardabil | IFA | 909 | 106 | 11.7 | Cross-sectional | Pregnant women | Moderate |
| **Yaghoub. et al [280]** | 2014 | West Azerbaijan | ELISA | 200 | 79 | 39.5 | Cross-sectional | Pregnant women | Low |
| **Davoodi. et al [281]** | 2011 | East Azarbaijan | CLIA | 200 | 82 | 41 | Cross-sectional | children | Low |
| **Davoodi. et al [282]** | 2012 | East Azarbaijan | ELISA | 200 | 73 | 36.5 | Cross-sectional | General | Low |
| **Jouyani. et al [283]** | 2019 | Ardabil | ELISA | 50 | 16 | 32 | Cross-sectional | General | Low |
| **Rezazadeh Varaghchi. et al [170]** | 2015 | South Khorasan | ELISA | 519 | 77 | 14.8 | Cross-sectional | General | Low |
| **Bitaraf. et al [284]** | 2017 | Tehran | ELISA | 3370 | 745 | 22.1 | Cross-sectional | blood donor | Low |
| **Kheirandish. et al [285]** | 2016 | Lorestan | ELISA | 170 | 65 | 38.2 | Case-control | General | Low |
| **Choubdarian. et al [286]** | 2019 | Kurdistan | ELISA | 200 | 29 | 14.5 | Case-control | General | Moderate |
| **Keighobadi. et al [287]** | 2021 | Mazandaran | ELISA | 90 | 79 | 87.8 | Case-control | General | Moderate |

**References**

1. Shahra M, Keshavarz H, Sahraeian MA, Shojaee S, Heidari A, Alimi R, et al. Associations between *Toxoplasma gondii* Infection and Multiple Sclerosis: A Case-Control Seroprevalence Study. Iran J Parasitol. 2023;18(2):165.

2. Rahnama M, Asgari Q, Petramfar P, Tasa D, Hemati V, Solgi R. The role of *Toxoplasma gondii* infection among multiple sclerosis patient compared to ordinary people in south of Iran: a case-control study. Mod Care J. 2020;17(3).

3. Sabzevari M, Tavalla M. Seroepidemiological Study of *Toxoplasma gondii* in Patients with Multiple Sclerosis in Ahvaz, Southeastern Iran. Med Lab J. 2017;11(3).

4. Saki J, Mowla K, Arjmand R, Kazemi F, Fallahizadeh S. Prevalence of *Toxoplasma gondii* and *Toxocara canis* Among Myositis Patients in the Southwest of Iran. Infectious Disorders-Drug Targets (Formerly Current Drug Targets-Infectious Disorders). 2021;21(1):43-48.

5. Mousavi M, Jamshidi A, Reisi JM. Serological study of toxoplasmosis among pregnant women of Nikshahr in 2012. RUJMS. 2014;21(123):45-53.

6. Saeedi M, Veghari GR, Marjani A. Seroepidemiologic evaluation of anti-*Toxoplasma* antibodies among women in north of Iran. Pakistan journal of biological sciences: PJBS. 2007;10(14):2359-62.

7. Pirali-Kheirabadi K, Tahmasby H, Manouchehri-Naeini K, Masoumi-Ghajari S. Serological survey of human *Toxoplasma gondii* infection in northern and central regions of Iran. Biol J Microorg. 2013;1(4):15-20.

8. Rostami A, Seyyedtabaei SJ, Aghamolaie S, Behniafar H, Lasjerdi Z, Abdolrasouli A, et al. Seroprevalence and risk factors associated with *Toxoplasma gondii* infection among rural communities in northern Iran. Revista do Instituto de Medicina Tropical de São Paulo. 2016;58.

9. Mehbod ASA SM, Ghorban K, Karamy M. seroepidemiology assay of toxoplasmosis in infants who was confined to bed in infants ward of Taleghani Hospital between 1979-80. AMHSR. 2005;3(11).

10. Kalantari N, Rezanejad J, Tamadoni A, Ghaffari S, Alipour J, Bayani M. Association between *Toxoplasma gondii* exposure and paediatrics haematological malignancies: a case–control study. Epidemiol *Infect*. 2018;146(15):1896-902.

11. Mahami Oskouei M, Hamidi F, Talebi M, Farhoudi M, Taheraghdam AA, Kazemi T, et al. The correlation between *Toxoplasma gondii* infection and Parkinson’s disease: a case-control study. J Parasit Dis. 2016;40:872-76.

12. Dalimiasl a, Arshad m. Sero-epidemiology of *Toxoplasma* Infection in PregnantWomen Referred to Al Zahra Hospital in Tabriz. JIUMS. 2012;20(3):55-62.

13. Ebrahimzadeh A, Mohammadi S, Salimi-Khorashad A, Jamshidi A. Seroprevalence of toxoplasmosis among pregnant women referring to the reference laboratory of Zahedan, Iran. IZJRMS. 2013;15(12).

14. Javadi EHS, Haghdoost M, Taghizadeh S, Oweysee H. *Toxoplasma* infection in pregnancy: Diagnosis and treatment. Int J Curr Res Acad Rev. 2014;2(8):274-80.

15. Soltani S, Ghaffari AD, Kahvaz MS, Sabaghan M, Pashmforosh M, Foroutan M. Detection of anti-*Toxoplasma* *gondii* IgG and IgM antibodies and associated risk factors during pregnancy in Southwest Iran. Infect Dis Obstet Gynecol. 2021;2021.

16. Saki J, Mohammadpour N, Moramezi F, Khademvatan S. Seroprevalence of *Toxoplasma gondii* in women who have aborted in comparison with the women with normal delivery in Ahvaz, southwest of Iran. World J. 2015;2015.

17. Cheraghi pour k, Sheikhian a, Maghsoud ah, Hejazi z, Rostami nejad m, moradi pour k. Seroprevalence study of toxoplasmosis in pregnant women referred to Aleshtar rural and urban health centers in 2008. YJMS. 2010;11(4):65-73.

18. Akhlaghi L, Tabatabaie F, Hadighi R, Maleki F, Hajialiani F, Dayer MS, et al. Diagnosis of acute toxoplasmosis in pregnant women referred to therapeutic centers of Alborz Province (Iran) using immunoglobulin G avidity ELISA technique. Asian Pac J Trop Dis. 2016;6(11):864-7.

19. Panah AS, Assadi M, Soufiani K, Barzegar G, Gharachorlou A, Emami Zeyd A. Seroprevalence of *Toxoplasma* *gondii* infection among pregnant women in Amol, Northern Iran. Life Sci J. 2013;10(2s):164-8.

20. Vakil N, Mosayebi M, Eslamirad Z. Susceptibility to Toxoplasmosis and its Risk Factors during Prenatal Care. Iran J Nurs. 2014;26(86):51-60.

21. Shieh M, Didehdar M, Hajihossein R, Ahmadi F, Eslamirad Z. Toxoplasmosis: seroprevalence in pregnant women, and serological and molecular screening in neonatal umbilical cord blood. Acta Trop. 2017;174:38-44.

22. Hariri SS, Heidari Z, Habibzadeh S, Shahbazzadegan S. Seroprevalence of *Toxoplasma* *gondii* among Pregnant Women in Ardabil, Iran (2021–2022). *Iran J Parasitol*. 2023;18(1):93.

23. Maani S, Kazemi M, Solhjoo K, Shadmand E, Rezanezhad H. Serological study of toxoplasmosis in pregnant women in Jahrom city, 2018. J Pars Univ Med Sci. 2022;18(2):1-8.

24. Motazedian MH, Fouladvand M, Barazesh A. The prevalence of toxoplasmosis in hemodialysis patients in Bushehr, Iran in 2012. J Mazandaran Univ Med Sci. 2016;26(141):123-30.

25. Dubey J, Murata F, Cerqueira-Cézar C, Kwok O, Su C. Economic and public health importance of *Toxoplasma* *gondii* infections in sheep: 2009–2020. Vet Parasitol. 2020;286:109195.

26. Nejad MR, Cheraghipour K, Mojard EN, Moradpour K, Razaghi M, Dabiri H. Seroprevalence and risk factors for *Toxoplasma* infection in a large cohort of pregnant women in Rural and Urban areas. HealthMED. 2011:338.

27. Norouzi LY, Sarkari B, Asgari Q, Khabisi SA. Molecular evaluation and seroprevalence of toxoplasmosis in pregnant women in Fars province, Southern Iran. Ann Med Health Sci Res. 2017;7(1):16-9.

28. Partoandazanpour A, sadeghi Dehkordi Z, nooshin a. Seroepidemiological study of toxoplasmosis in pregnant women of Qorve city. J Zoonotic Dis 2:31.

29. Shirdel S, Sharbatkhori M, Pagheh AS, Dadimoghadam Y, Soosaraie M, Gholami S. Seroepidemiology of *Toxoplasma* *gondii* infection in pregnant women and risk factors of the disease in Golestan province, Iran. J Mazandaran Univ Med Sci. 2017;27(152):63-71.

30. Salehi M, Niazkar HR, Mahmoudian A, Nezami H, Emamdadi S, Naseri P, et al. Seroepidemiological Survey of Toxoplasmosis Among Pregnant and Abortive Women of Gonabad. Crescent J Med Biol. 2021;8(2).

31. Dadimoghdam Y, Anemohamadzadeh S, Rastegar N. Seroprevalence of Toxoplasmosis in pregnant women referred to the Women\’s Clinic Social Security Hospital, Gonbad kavoos city, Golestan province. 7th Int Congress SID*.* 2014.

32. Sharbatkhori M, Moghaddam YD, Pagheh AS, Mohammadi R, Mofidi HH, Shojaee S. Seroprevalence of *Toxoplasma* *gondii* infections in pregnant women in Gorgan city, Golestan province, northern Iran-2012. Iran J Parasitol. 2014;9(2):181.

33. Falah M, Matini M, Taherkhani HA, Rabiei S, Hajiloui M. Seroepidemiology of toxoplasmosis in first trimester pregnant women in Hamadan city in 2013-2015. Avicenna J Clin Med*.* 2006; 13 (1) :33-37.

34. Maghsood A, Fallah M, Moradi H, Shanazi H, Moradi Y. Seroepidemiology of toxoplasmosis in pregnant women referring to health centers in the city of Hamadan in 1391. Pajouhan Sci J. 2013;11(2):3-8.

35. Abdi J, Shojaee S, Mirzaee A, Keshavarz H. Seroprevalence of Toxoplasmosis in Pregnant Women in Ilam Province, Iran. Iran J Parasitol. 1970;3(2).

36. Rezanezhad SMMKKSESH. Serological study of toxoplasmosis in pregnant women in Jahrom city, 2018. *PJMS*. 2018;18:1-8. doi: 10.52547/jmj.18.2.1.

37. Akhlaghi L, Ghasemi A, Hadighi R, Tabatabaie F. Study of seroprevalence and risk factors for *Toxoplasma* *gondii* among pregnant women in Karaj township of Alborz province [2013]. J Entomol Zool Stud*.* 2014;2(6):217-9.

38. Seroepidemiology of toxoplasmosis in pregnant women referred to the Kashan Simulated Maternity Hospital, 1990-1993. Pejouhesh dar Pezeshki (Research in Medicine). 2001;25(4):243-8.

39. Bahare Zalei DP, Soraya bilvaye, Haleh Jalilzadeh hamidi. Seroepidemiology of Toxoplasmosis in Pregnant Women Referred to Reference laboratory in Kermanshah, 2014-2015. J Clin Res Paramed Sci. 2016;5(2).

40. Talari SA, Hejazi SH, Rasti S, Shadzi S. Seroepidemiology of *Toxoplasma* *Gondii* in pregnant females referring to Ashrafi Isfahani Hospital in Khomeinishahr, 1998-2000. Feyz Med Sci J. 2003;6(4):32-7.

41. Cheraghipour K, Taherkhani H, Fallah M, Sheikhian A, Sardarian K, Rostami Nejad M, et al. Seroprevalence of Toxoplasmosis in Pregnant Women Admitted to the Health Centers of Khorram-Abad City, Iran. Avicenna J Clin Med. 2010;17(3):46-51.

42. Sharifi K, Farash BRH, Fatemeh T, Khaledi A, Sharifi K, Shamsian SAA. Diagnosis of acute toxoplasmosis by IgG and IgM antibodies and IgG avidity in pregnant women from Mashhad, Eastern Iran. Iran J Parasitol. 2019;14(4):639.

43. Hoseini SA, Dehgani N, Sharif M, Daryani A, Gholami S, Ebrahimi F, et al. Serological survey of toxoplasmosis in pregnant women. Journal of Mazandaran University of Medical Sciences. 2014;24(114):146-50.

44. Mohaghegh MA, Kalani H, Hashemi M, Hashemi S, Yazdnezhad SK, Hejazi SH, et al. Toxoplasmosis-related risk factors in pregnant women in the North Khorasan province, Iran. Int J Med Res Health Sci*.* 2016;5(8):370-4.

45. Aliabadi J, Khamesi S, Ghorbanpour H, Rahimi Esboei B. Seroprevalence of IgG, IgM and IgA against *Toxoplasma* *gondii* in pregnant women in first trimester in northern Khorasan Province, Iran. *J North Khorasan Univ Med Sci.* 2017;9(2):243-52. doi: 10.18869/acadpub.jnkums.9.2.243.

46. Eskandarian A. Seroepidemiology of toxoplasmosis in admitted pregnant women in maternity ward of Kowsar teaching and cure center in Qazvin-2006. Iran J Med Microbiol. 2009;3(2):73-9.

47. Maleki F, Tabatabaie F, Falahati M, Akhlaghi L, Shemshad K. Comparison of Toxoplasmosis frequency in pregnant women during two years in Qom province (iran). HealthMED. 2013:451.

48. Haeri MR, Jalalizadegan B, Tabatabaie F. Recognition of acute toxoplasmosis with IgG avidity ELISA test in the pregnant women (the first trimester) in Qom Province, Iran, during two years (2012-2013). Am J Life Sci. 2014;2(6-3):18-21. doi: 10.11648/j.ajls.s.2014020603.14

49. Fallahizadeh S, Jelowdar A, Kazemi F, Cheraghian B. Seroprevalence of anti-*toxoplasma* IgG and IgM among pregnant women of Shush county, southwest of Iran. Int J Infect. 2018;5(1).

50. Rahmati-Balaghaleh M, Hosseini Farash BR, Zarean M, Hatami-Pourdehno S, Mirahmadi H, Jarahi L, et al. Diagnosis of acute toxoplasmosis by IgG avidity method in pregnant women referred to health centers in south-eastern Iran. J Parasit Dis. 2019;43:517-21.

51. Pashaie Naghadeh A, Dabirzadeh M, Davoodi T, Hashemi M. Seroepidemiology of Toxoplasmose in Pregnant Women in Tabas City, Iran. mljgoums. 2015;9.

52. Ahmadpour E, Zargami E, Mahami-Oskouei M, Spotin A, Shahbazi A, Kafil HS, et al. Diagnosis of *Toxoplasma* *gondii* infection in pregnant women using automated chemiluminescence and quantitative real time PCR. Asian Pac J Trop Med. 2019;12(1):26-31.

53. Ghasemloo H, Ghomashlooyan M, Hooshyar H. Seroprevalence of *Toxoplasma* *gondii* infection among pregnant women admitted at Shahid Akbar Abadi hospital, Tehran, Iran, 2010-2013. J Med Microbiol Infect Dis. 2014;2(1):16-8.

54. Gharavi M. Seroepidemiological survey of toxoplasmosis in pregnant women in Tehran. Hakim Res J. 2002;5(2):91-98.

55. Mozaffari M, Mozafari M, Otoukesh M, Ghaemi M, Mohebbi M. Investigation of *Toxoplasma* *gondii* in Pregnant Women: A Strategy for Personalized Medicine. Personal Med J. 2019;4(13):19-22.

56. Hazrati Tappeh K, Mousavi SJ, Bouzorg Omid A, Ali Nejad V, Alizadeh H. Seroepidemiology and risk factors of toxoplasmosis in pregnant women in Urmia city. St Med Sci J. 2015;26(4):296-302.

57. Khameneh ZR, Hanifian H, Rostamzadeh A. Seroprevalence of Toxoplasmosis in Pregnant Women in Urmia, Iran. Int J Enteric Pathog. 2016;4(2):3-33350.

58. Mohammadnejad AE, Eslami G, Shamsi F, Pirnejad A, Samie A, Safabakhsh J, et al. Prevalence of Food-Borne *Toxoplasma* in Pregnant Women Population of Urmia, Iran. J Food Qual Hazards Control. 2018;5(1).

59. Raissi V, Taghipour A, Navi Z, Etemadi S, Sohrabi Z, Sohrabi N, et al. Seroprevalence of *Toxoplasma* *gondii* and *Toxocara* spp. infections among pregnant women with and without previous abortions in the west of Iran. J Obstet Gynaecol Res. 2020;46(3):382-8.

60. AnvariTafti M, Ghafourzadeh M. Seroepidemiology of *Toxoplasma* infection in pregnant women in Yazd in 2012. Tolooebehdasht. 2014;13(3):116-25.

61. M Sbhssmn. Investigation of The Prevalence of *Toxoplasma* Antibodies in Women During Pregnancy in Child and Mother's clinic in Zahedan.. IJOGI 2000;3(6-5):72-75.

62. Ebrahimzadeh A, Mohammadi S, Davoodi T, Salimi Khorashad A, Jamshidi A. Seroepidemiology of Toxoplasmosis among Pregnant Women Referring to the Reference Laboratory of Zahedan, Iran (2011). Med Lab. 2013;7(3):61-68.

63. Sharifi-Mood B, Hashemi-Shahri M, Salehi M, Naderi M, Naser-Poor T. Seroepidemiology of *Toxoplasma* infection in the pregnant women in Zahedan, Southeast of Iran. J Res Health Sci. 2011;4(2):1-3.

64. Kareen Aaln. Prevalence of *Toxoplasma* Antibodies in Pregnant Women Referred to Shariati Hospital Bandar Abbas, Iran 1999-2000. Hormozgan Med J*.* 2002;6(4):25-30.

65. Firouz ZE, Kaboosi H, Nasiri AF, Tabatabaie SS, Golhasani-Keshtan F, Zaboli F. A comparative serological study of toxoplasmosis in pregnant women by CLIA and ELISA methods in Chalus City Iran. Iran Red Crescent Med. 2014;16(4).

66. Babaie J, Amiri S, Mostafavi E, Hassan N, Lotfi P, Esmaeili Rastaghi AR, et al. Seroprevalence and risk factors for *Toxoplasma gondii* infection among pregnant women in Northeast Iran. Clin Vaccine Immunol. 2013;20(11):1771-73.

67. Zavari T, Mirzaei S, Rezaeian M, Zainodini N, Zare-Bidaki M. A Study of the Seroprevalence of IgG and IgM Immunoglobulins Against *Toxoplasma gondii* in Pregnant Women Referred to Niknafs Maternity Ward of Rafsanjan City and in Umbilical Cord of their Infants in 2013. JRUMS. 2015;14(6):507-18.

68. Sadeghi Dehkordi Z, Partoandazanpour A, Adolmaleki N. Seroprevalence and risk factors of *Toxoplasma gondii* infection among pregnant women in Sanandaj, west of Iran: A Cross-Sectional Study. J Zoonotic Dis. 2022;6(2):78-83.

69. Ahmadpour GR, Ezatpour B, Hadighi R, Oormazdi H, Akhlaghi L, Tabatabaei F, et al. Seroepidemiology of *Toxoplasma gondii* infection in pregnant women in west Iran: determined by ELISA and PCR analysis. J Zoonotic Dis. 2017;41:237-42.

70. Hajsoleimani F, Ataeian A, Nourian A, Mazloomzadeh S. Seroprevalence of *Toxoplasma gondii* in pregnant women and bioassay of IgM positive cases in Zanjan, Northwest of Iran. Iran J Parasitol. 2012;7(2):82.

71. Akhlaghi L, Shirbazou S, Maleki F, Keyghobadi A, Tabaraei Y, Tabatabaie F. Seroepidemiology of *Toxoplasma* infection in pregnant women in Qom Province, Iran (2010). Life Sci J. 2013;10(SUPPL.):322-25.

72. Manouchehri naeeni k, keshavarz h, abdizade dehkordi r, zebardast n, kheiri s, khalafian p, et al. Seroprevalence of anti-*Toxoplasma* antibodies among pregnant women from Chaharmahal and Bakhtyari province using indirect immunoflurescent in 2006-2007. JSUMS. 2007;8(4):74-80.

73. Jahantigh FF, Rasekh M, Ganjali M, Sarani A. Seroprevalence of *Toxoplasma gondii* infection among pregnant women and small ruminant populations in Sistan region, Iran. Iran J Vet Med. 2020;14(3).

74. Noorbakhsh S, Mamishi S, Rimaz S, Monavari M. Toxoplasmosis in primiparus pregnant women and their neonates. Iran J Publ Health 2002.

75. Khademi SZ, Ghaffarifar F, Dalimi A, Davoodian P, Abdoli A. Prevalence and risk factors of *Toxoplasma gondii* infection among pregnant women in Hormozgan Province, South of Iran. Iran J Parasitol. 2019;14(1):167.

76. Nourollahpour Shiadeh M, Rostami A, Pearce B, Gholipourmalekabadi M, Newport DJ, Danesh M, et al. The correlation between *Toxoplasma gondii* infection and prenatal depression in pregnant women. Eur J Clin Microbiol Infect Dis. 2016;35:1829-35.

77. Fazeli Z, Nazemalhosseini Mojarad E, Pourhoseingholi MA, Rostami K, Barzegar F, Zali MR. Prevalence of celiac disease and toxoplasmosis during pregnancy. Med Sci J Islamic Azad Univ Tehran Med Branch. 2013;22(4):288-93.

78. Abdollahian E, Shafiei R, Mokhber N, Kalantar K, Abdolmajid F. Seroepidemiological study of *Toxoplasma gondii* infection among psychiatric patients in Mashhad, Northeast of Iran. Iran J Parasitol. 2017;12(1):117.

79. Soleymani E, Azimi A, Faizi F, Kordi S, Azorde M, Abedian R, et al. Seroprevalence of *Toxoplasma gondii* in the Rural Population of Qaemshahr, Northern Iran in 2019. Avicenna J Clin Microbiol Infect. 2021;8(1):34-8.

80. director MSs, Hashemi HJ. Serological prevalence of *Toxoplasma gondii* in girls referred to Qazvin community medical center for prenatal tests (2008). JQUMS*.* 2012.

81. Hamza S, Shakib J, religion S, Roshan P, Majidi H. Seroepidemiology of toxoplasmosis in patients visiting medical diagnostic laboratories in Ramsar city in 2016. J Guilan Univ Med Sci. 2023;32(2):106-15.

82. Rasti S, Hassanzadeh M, Soliemani A, Hooshyar H, Mousavi SGA, Nikoueinejad H, et al. Serological and molecular survey of toxoplasmosis in renal transplant recipients and hemodialysis patients in Kashan and Qom regions, central Iran. Ren Fail. 2016;38(6):970-3.

83. Soltani S, Khademvatan S, Saki J, Shahbazian H. Detection of toxoplasmosis in renal transplant recipients by ELISA and PCR methods in Ahvaz, south-west of Iran. Jundishapur J Microbiol. 2013;6(9).

84. Kawakb P, vala Mh, Taqvai MJ, Haghigi S. Determining the rate of toxoplasmosis infection in kidney transplant recipients and donors 2009. IJIDTM. 2009;47:43-5.

85. Zarean M, Mastroeni P, Moghaddas E, Farash BRH, Raouf-Rahmati A, Jamali J, et al. Toxoplasmosis frequency rate in rheumatoid arthritis patients in Northeastern Iran. Iran J Parasitol. 2022.

86. Mojadadi M-S, Mahmoodabadi N, Sajadiniya Z, Golmohamadi R, Elyasi H. A Seroepidemiological Study of Toxoplasmosis Among Female Students of Sabzevar University of Medical Sciences. JSUMS. 2016;23(3):490-5.

87. Mohammadi P, Tahirpour A, Mohammadi H. Seroepidemiology of toxoplasmosis in women referring to premarital counseling center in Sanandaj city in 2015. IJIDTM. 2015;40:25-29.

88. Sharif M, Daryani A, Barzegar G, Nasrolahei M. A seroepidemiological survey for toxoplasmosis among schoolchildren of Sari, Northern Iran. Trop Biomed. 2010;27(2):220-25.

89. Alipour A, Shojaee S, Mohebali M, Tehranidoost M, Masoleh FA, Keshavarz H. *Toxoplasma* infection in schizophrenia patients: a comparative study with control group. Iran J Parasitol. 2011;6(2):31.

90. Banihashem SS, Saber FY, Motazedian S, Mardani M, Shamsi A, Nazari M, et al. Serologic evaluation of *cytomegalovirus* (CMV), *Toxoplasma gondii* and *Brucella* in schizophrenia patients. Caspian J Intern Med. 2023;14(3):560.

91. Khademvatan S, Khajeddin N, Izadi S, Yousefi E. Investigation of anti-*Toxocara* and anti-*Toxoplasma* antibodies in patients with schizophrenia disorder. Schizophr Res Treatment. 2014;2014.

92. Ebadi M, Akhlaghi H, Zamani MM, Beheshti H, Abolhassani H, Ayadi A, et al. The correlation between *Toxoplasma* *gondii* infection and schizophrenia: A comparative study with family members (control group). Scimetr. 2014;2(1).

93. Ansari‐Lari M, Farashbandi H, Mohammadi F. Association of *Toxoplasma gondii* infection with schizophrenia and its relationship with suicide attempts in these patients. Trop Med Int Health. 2017;22(10):1322-7.

94. Meftahi B, Abdollahian E, Nematollahi A, Razmi G. A Study of Association of *Toxoplasma gondii* Infection With Schizophrenia in Mashhad Area, Khorasan Razavi Province, Iran. Int J Epidemiol Res,[online]. 2021;8(2):88-92.

95. Daryani A, Sharif M, Hosseini SH, Karimi SA, Gholami S. Serological survey of *Toxoplasma gondii* in schizophrenia patients referred to Psychiatric Hospital, Sari City, Iran. Trop Biomed. 2010;27(3):476-82.

96. Babaie J, Sayyah M, Gharagozli K, Mostafavi E, Golkar M. Seroepidemiological study of *Toxoplasma gondii* infection in a population of Iranian epileptic patients. EXCLIJ. 2017;16:256.

97. Taghizadeh H, Shahriarirad R, Erfani A, Nekouei F, Seifbehzad S, Khabisi S, et al. Seroepidemiological survey of toxoplasmosis among female university students in Shiraz, southern Iran. Ann Trop Med Public Health. 2017;10(2):362-.

98. Makiani MJ, Davoodian P, Golsha R, Dehghani M, Rajaee M, Mahoori K, et al. Seroepidemiology and risk factors of toxoplasmosis in the first trimester among pregnant women. Dis Diagn. 2012;1(2):12-7.

99. Rajaii M, Pourhassan A, Asle-Rahnamaie-Akbari N, Aghebati L, Xie JL, Goldust M, et al. Seroepidemiology of toxoplasmosis in childbearing women of Northwest Iran. Infez Med. 2013;21(3):194-200.

100. Ramezani M, Shojaii M, Asadollahi M, Karimialavijeh E, Gharagozli K. Seroprevalence of *Toxoplasma gondii* in Iranian patients with idiopathic Parkinson's disease. Clin Exp Neuroimmunol. 2016;7(4):361-5.

101. Arbabi M, Talari SA. The prevalence of Toxoplasmosis in subjects involved in meat industry and pregnant women in Kashan. Feyz Med Sci J. 2002;6(2):28-38.

102. Torkan S, Momtaz H, Abdizadeh R. Comparison of Toxoplasmosis prevalence in individuals with and without cat contact in Isfahan using indirect Immunofluorescent. JSUMS. 2008;10(3):83-89.

103. Manochehri NK, Mortazaee S, Zebardast N, Kheiri S. Seroprevalence and some of risk factors affecting *Toxoplasma infection* in Chaharmahal va Bakhtiyari province, Iran. JSUMS. 2012.

104. Shaddel M, Mirzaii-Dizgah I, Hoshangi M. Anti-*Toxoplasma gondii* antibody levels in blood supply of Shiraz Blood Transfusion Institute, Iran. Iran J Parasitol. 2014;9(1):120.

105. Razavi SM, Kayseri H, Ashari HE. Seroepidemiology of toxoplasmosis with IFA method in students of veterinary faculty of Shiraz University. J Vet Res. 2002;2.

106. Dorri M, Dabirzadeh M, Maroufi Y, Afshari M, Chokamy MB. Prevalence of anti-*Toxoplasma* IgG and IgM in hemodialysis patients comparing to healthy individuals in Sistan area, Iran. J Nephropharmacol. 2017;6(2):106-9.

107. Youssefi M, Khadem-Rezaiyan M, Azari-Garmjan G-A, Jarahi L, Shamsian A-A, Moghaddas E. Prevalence of *Toxoplasma* and *Echinococcus* IgG antibodies in slaughterhouse workers, a serosurvey in Northeast Iran. Annal Parasitol. 2018;64(4).

108. Salahi-Moghaddam A, Hafizi A. A serological study on *Toxoplasma* *gondii* infection among people in south of Tehran, Iran. The Korean J Parasitol. 2009;47(1):61.

109. Soltani S, Foroutan M, Afshari H, Hezarian M, Kahvaz MS. Seroepidemiological evaluation of *Toxoplasma gondii* immunity among the general population in southwest of Iran. J Parasit Dis. 2018;42(4):636-42.

110. Nematollahi S, Hajimohammadi B, Eslami G, Ehrampoush MH, Tafi AD. Prevalence and risk factors of Toxoplasmosis among Women of Reproductive age, Southwestern Iran. J Egypt Soc Parasitol. 2022;52(2):341-8.

111. Kheirandish F, Ezatpour B, Fallahi S, Tarahi MJ, Hosseini P, Rouzbahani AK, et al. *Toxoplasma* serology status and risk of miscarriage, a case-control study among women with a history of spontaneous abortion. Int J Fertil Steril. 2019;13(3):184.

112. Sadaghian M, Jafari R. Prevalence of *Toxoplasma* infection in veterinary laboratory sciences students comparing to ordinary people: a case–control study. J Parasit Dis. 2016;40:768-71.

113. Saki J, Eskandari E, Feghhi M. Study of toxoplasmosis and toxocariasis in patients suffering from ophthalmic disorders using serological and molecular methods. Int Ophthalmol. 2020;40:2151-7.

114. Jafari R, Sadaghian M, Safari M. Seroprevalence of *Toxoplasma* *gondii* infection and related risk factors in Tabriz city, Iran, 2008. J Res Health Sci. 2012;12(2):119-21.

115. Fatollahzadeh M, Jafari R, Mohammadi F, Ghayemmaghammi N, Rezvan S, Parsaii M, et al. Study of anti-*Toxoplasma* IgG and IgM seropositivity among subjects referred to the central laboratory in Tabriz, Iran, 2013-2014. Avicenna J Clin Microbiol Infect. 2016;3(3):35975.

116. Nasser RA, Behzad G, Hadi H, Ramin P. Investigating the diagnostic value of *Toxoplasma gondii*-specific IgA antibodies with *Toxoplasma*-specific IgM and IgG antibodies in pregnant women in Tabriz. J Doc Lab. 2005.

117. Ghadamgahi F, Bahadoran M, Shariat-Bahadori E, Ahmadi-Ahvaz N, Ghadrdoost B, Hejazi SH. Study of Serological Toxoplasmosis and Risk Factors Associated with Infection in Women Referred to Labs of Northern Tehran, Iran. J Isfahan Med Sch. 2013;31(248):1257-66.

118. Shariat E, Dalir Ghaffari A, Mosavipoor S, Namroodi S, Sadraie J. Electrochemiluminescence Epidemiologic Detection of *Toxoplasma* *gondii* Infection in Pregnant Women With Direct and Indirect Diagnostic Techniques (ELISA Avidity Plus Biochemical Assay), Tehran, Iran. Epidemiol Health System J. 2019;6(2):49-54.

119. Moghimi M, Doosti M, Vahedian-Ardakani H, Talebi A, Akhavan-Ghalibaf M, Najafi A, et al. Serological study on cytomegalovirus and *Toxoplasma gondii* in thalassemia major patients of Yazd, Iran. Iran J Ped Hematol Oncol. 2015;5(3):149.

120. Yousefi E, Foroutan M, Salehi R, Khademvatan S. Detection of acute and chronic toxoplasmosis amongst multi-transfused thalassemia patients in southwest of Iran. J Acute Dis. 2017;6(6):120-5.

121. Hanifehpour H, Shariat SKS, Ghafari MS, Kheirandish F, Saber V, Fallahi S. Serological and molecular diagnosis of *Toxoplasma gondii* infections in thalassemia patients. Iran J Parasitol. 2019;14(1):20.

122. Soltani S, Tavakoli S, Sabaghan M, Kahvaz MS, Pashmforosh M, Foroutan M. The probable association between chronic *Toxoplasma* *gondii* infection and type 1 and type 2 diabetes mellitus: a case-control study. Interdiscip Perspect Infect Dis. 2021;2021:1-6.

123. Mohammadpour A, Keshavarz H, Mohebali M, Salimi M, Teimouri A, Shojaee S. The relation of serum prolactin levels and *Toxoplasma* infection in humans. Int J Gen Med. 2018:7-12.

124. Rasti S, Ghasemi FS, Abdoli A, Piroozmand A, Mousavi SGA, Fakhrie‐Kashan Z. ToRCH “co‐infections” are associated with increased risk of abortion in pregnant women. Congen Anomal. 2016;56(2):73-78.

125. Arefkhah N, Sarkari B, Rozrokh S, Rezaei Z, Moshfe A. Toxoplasmosis in Nomadic Communities: A Seroepidemiological Study in Southwestern Iran. Ann Ig. 2020;32(1).

126. Ghasemi FS, Rasti S, Piroozmand A, Bandehpour M, Kazemi B, Mousavi SGA, et al. Toxoplasmosis-associated abortion and stillbirth in Tehran, Iran. J Matern-Fetal Neonatal Med. 2016;29(2):248-51.

127. Soltani S, Tavakoli S, Barati M, Pashmforosh M, Foroutan M. The seroprevalence of *Toxoplasma gondii* in patients with type 2 diabetes mellitus: a case-control study in Southwest Iran. Mil Caring Sci. 2020;7(3):252-60.

128. Kalantari M, Asgari Q, Rostami K, Naderi S, Mohammadpour I, Yousefi M, et al. Molecular and serological evaluation of *Toxoplasma* *gondii* among female university students in Mamasani district, Fars province, southern Iran. J Health Sci Surveill Syst. 2020;8(2):75-80.

129. Farhang HH, Ezatzadeh A, Anzabi Y. Prevalence of *Toxoplasma Gondii* Antibodies in the Serum of Urban Residents and Ranchers in Tabriz, Iran. Crescent J Med Biol Sci. 2014;1(3):85-9.

130. Rasouli S, Khodadadi A, Khodadadi M. Seroepidemiologic study of toxoplasmosis incidence in Urmia population by using the Electrochemiluminescence Immunoassay (ECLIA). Comp Pathobiol. 2010;6(4):115-22.

131. Sadaghian M, Amani S, Jafari R. Prevalence of toxoplasmosis and related risk factors among humans referred to main laboratories of Urmia city, North West of Iran, 2013. J Parasit Dis. 2016;40:520-23.

132. Tappeh KH, Musavi J, Safa MB, Galavani H, Alizadeh H. Prevalence of IgG and IgM anti-*Toxoplasma gondii* antibodies in blood donors at Urmia blood transfusion organization, Iran. Turkiye Parazitol Derg. 2017;41(1):1.

133. Kashan ZF, Shojaee S, Keshavarz H, Arbabi M, Delavari M, Salimi M. Vitamin D deficiency and *Toxoplasma* infection. Iran J Public Health. 2019;48(6):1184-86.

134. Mousavi-Hasanzadeh M, Sarmadian H, Ghasemikhah R, Didehdar M, Shahdoust M, Maleki M, et al. Evaluation of *Toxoplasma gondii* infection in western Iran: seroepidemiology and risk factors analysis. Trop Med Health. 2020;48:1-7.

135. Rahimkhani M, Kazemian K, Zarebavani M, Khavarydaneshvar A, Safari M. Seroprevalence of *Toxoplasma* IgG and IgM antibodies in Iranian Young Women before Pregnancy. JTAS. 2021;(6):1-5.

136. Saeidi M. Seroepidemiology of anti-*Toxoplasma* antibodies in women who refered for marriage consultation. J Gorgan Univ Med Sci. 2003;9:64-71.

137. Bafghi AF, Bakhi SS, Eslami G, Vakili M. Seroprevalence of *Toxoplasma Gondii* Infection among Couples in the Verge of Marriage. J Tolooe behdasht. 2021;19(5).

138. Bokharaei-Salim F, Khanaliha K, Sayyahfar S, Sadeghi M, Tavakoli A, Salemi B. Seroprevalence and Molecular Investigation of Toxoplasmosis Among Working Children in Tehran. Arch Pediatr Infect Dis. 2023;11(1).

139. Ghorbani A, Niya A, Khatayi G, Mohammadi R, Malekzadeh J, KanaanGorjipour AAM, et al. Determining seroprevelence of *Toxoplasma* *gondii* in girls referring to counseling centers before marriage in the city of Yasuj in the year 1391. Life Sci J. 2013;10(12s).

140. Fattahi BA, Anvari R, Anvari MH. Toxoplasmosis Seroepidemiology in Serum of Suspected Patients Attending Medical Lab, in 2013. J Community Health Res*.* 2015.

141. Fattahi Bafghi A, Sadeghi Bakhi S, Vakili M. Seroprevalence of *Toxoplasma gondii* infection among young couples near marriage referred to prenatal counseling in Yazd city, central of Iran. J Tolooe behdasht. 2020;19(5):33-43.

142. Hajipour N, Hassanzadeh P. Seroprevalence of IgG and IgM antibodies against *Toxoplasma gondii* in pre-pregnancy women in Yazd, Iran. J Zoonotic Dis. 2023.

143. Gharavi MJ, Roozbehani M, Miahipour A, Oshaghi M, Gharegozlou B, Kalantar E, et al. Prevalence of anti-*Toxoplasma gondii* antibodies in young Iranians: The Caspian III study. Arch Pediatr Infect Dis. 2018;6(1).

144. Haghighi JD, Hosseini A, Shafiei R, Mehravaran A, Alijani E, Mirahmadi H. Evaluation of *Toxoplasma gondii* antibodies in addicted and non-addicted women in Zahedan, Southeast of Iran. Int J High Risk Behav Addict. 2020;9(3).

145. Khabisi SA, Almasi SZ, Zadeh SL. Seroprevalence and Risk Factors Associated with *Toxoplasma gondii* Infection in the Population Referred to Rural and Urban Health Care Centers in Zahedan, Primary Referral Level, in Southeastern Iran. J Parasitol Res. 2022;2022.

146. Jafari-Modrek M, Hasanzadeh R, Azizi H, Hatam-Nahavandi K. A Seroprevalence Study of Toxoplasmosis in Female Students in Zahedan, South East of Iran. Iran J Public Health. 2019;48(5):988.

147. Sardarian K, Maghsood A, Farimani M, Hajilooi M, Saidijam M, Ghane ZZ, et al. Detection of *Toxoplasma gondii* B1 Gene and IgM in IgG Seropositive Pregnant Women. J Clin Lab. 2019;65.

148. Soltani S, Moghaddam H, Fatemeh M, Tavakoli S. The Seroprevalence of Anti-*Toxoplasma* *Gondii* IgG and IgM Antibodies in Serum Samples Referred to Educational Hospitals of Abadan. J Isfahan Med Sch. 2000;40(692):843-50. doi: 10.48305/jims.v40.i692.0843.

149. Maraghi S, YadYad M, Sheikhi M, LatifiS M. Frequency of Anti-*Toxoplasma* Antibodies in Midwifery and Nursing Students of Abadan Islamic AzadUniversity Students in 2011. Armaghane Danesh. 2013;18(4):327-36.

150. Sotoudeh AR. Anti-*Toxoplasma* antibody in women with a history of miscarriage or stillbirth. *J Jahrom Univ Med Sci*. 2007;4(4):47-52.

151. Saki J, Zamanpour M, Najafian M, Mohammadpour N, Foroutan M. Detection of acute and chronic *Toxoplasma gondii* infection among women with history of abortion in the Southwest of Iran. J Parasitol Res. 2021;2021.

152. Shahnaz AB, Majid EH, Elham N, Salari Z. Comparison of the presence of anti-*toxoplasma* antibodies in pregnancies leading to abortion and normal pregnancies. IJOGI. 2012;14(1):1-6.

153. Ebadi P, Solhju K, Eftekhari F. Determining the prevalence of anti-*toxoplasma* antibodies in women with repeated spontaneous abortions compared to women with normal delivery. J Pars Univ Med Sci. 2011;9(1):33-7.

154. Ziyai Kajbaf Tahereh TMs-. Investigation of the frequency of positive serology against *Toxoplasma* among women of reproductive age in Ahvaz city in 2012. Jundishapour Med Sci J. 2008;7(56):92-9.

155. Afrogh P. Investigation of the prevalence of IgG and IgM anti-*toxoplasma* antibodies in girls about to get married who referred to health centers in Ahvaz and suburbs in 1385. Khuzestan Prov Unit. 2007.

156. Sohrabi A, Samarbafzadeh A, Makvandi M, Maraghi S, Razi T, Darban D. A seroepidemiological study of Parvovirus B19, *Toxoplasma* *gondii* and *Chlamydia trachomatis* in pregnant women referring to Obs & Gyn ward of Ahwaz Imam Khomeini Hospital. J Reprod Infertil. 2007;8(2).

157. Rahaa F. Seroepidemiology of Toxoplasmosis among girls studentsAhvaz Joundishapoor University Of Medical Sciences . Iran J Infect Dis Trop Med*.* 2005;10(31):35-41.

158. Fallah E, Rasuli A, Shahbazi A, Ghojazadeh M, Khanmohammadi M, Hamzavi F, et al. Seroprevalence of *Toxoplasma* *gondii* infection among high school girls in Ajabshir from East Azarbaijan province, Iran. J Caring Sci. 2014;3(3):205.

159. Shahighi M, Heidari A, Keshavarz H, Bairami A, Shojaee S, Sezavar M, et al. Seroepidemiological study of toxoplasmosis in women referred to a pre-marriage counseling center in Alborz Province, Iran. BMC Res Notes. 2021;14:1-6.

160. Mahami-Oskouei M, Hamidi F, Talebi M, Farhoudi M, Taheraghdam AA, Kazemi T, et al. Toxoplasmosis and Alzheimer: can *Toxoplasma* *gondii* really be introduced as a risk factor in etiology of Alzheimer? Parasitol Res. 2016;115:3169-74.

161. Rashno MM, Fallahi S, Kheirandish F, Bagheri S, Kayedi M, Birjandi M. Seroprevalence of *Toxoplasma gondii* infection in patients with Alzheimer’s disease. Arch Clin Infect Dis. 2016.

162. Mohammadi A, Shojaee S, Salimi M, Zareei M, Mohebali M, Keshavarz H. Seroepidemiological study of toxoplasmosis in women referred to arak marriage consulting center during 2012–2013. Iran J Public Health. 2015;44(5):654.

163. Dariani E, Sagha M. Sero-epidemiology of toxoplasmosis in girls referred of Ardabil heath center for pre-marriage tests. J Ardabil Univ Med Sci. 2004;4(13):19-24.

164. Alimohammadi H, Fouladi N, Amani F. Seroepidemiology of toxoplasmosis in women based on tests before marriage. J Ardabil Univ Med Sci. 2008;8(4):408-13.

165. Afsharpaiman S, Skandari A, Maryam ZJ, Radfar S, Shirbazoo S, Amirsalari S, et al. An assessment of Toxoplasmosis antibodies seropositivity in children suffering Autism. Tehran Univ Med J. 2014;72(2):106-12.

166. oshra Azizy NH, Hossein Hamidynejat. Study the Relationship Between *Toxoplasma gondii* Infection and Autism Disorder in Children. J Vet Res. 2020;75(4):413-7. doi: 10.22059/jvr.2019.252512.2764.

167. Hamid N, Azizy B, Hamidynejat H. Comparison of the infection of *Toxoplasma gondii* and aggression in autism and normal children. Sadra Med J. 2020;8(3):249-62.

168. Kalantari N, Ghaffari S, Bayani M, Nouri M, Yahyapour Y. Evaluation of seroprevalence and seroconversion rates of *Toxoplasma gondii* infection in female students at Babol University of Medical Sciences, Babol, Iran. Caspian J Reprod Med. 2017;3(1):12-7.

169. Kanani B, Namaei MH, Kareshk AT, Solgi R. Seroprevalence of *Toxoplasma gondii* infection among women of reproductive age in Birjand, Iran. Mod Care J. 2022;19(4).

170. Rezazadeh Varaghchi J, Ahmadi Shadmehri A, Ahmadi Shadmehri A. Seroepidemiology of toxoplasmosis in women referred to Birjand Milad genetic counseling center during 2011-2013. J Kerman Univ Med Sci. 2015;22(5):524-32.

171. Davami MH, Pourahmad M, Baharlou R, Jahromi AS, Vasmejani AA, Solhjoo K, et al. Seroepidemiology of *Toxoplasma* infection in blood donors in Jahrom District, Southern Iran. Asian Pac J Trop Biomed. 2015;5(12):1060-4.

172. Sarkari B, Shafiei R, Zare M, Sohrabpour S, Kasraian L. Seroprevalence and molecular diagnosis of *Toxoplasma gondii* infection among blood donors in southern Iran. J Infect Dev Ctriess. 2014;8(04):543-7.

173. Asfaram S, Rezaei R, Fakhar M, Ghezelbash B, Nakhaei M, Hezarjaribi HZ, et al. High occurrence of *Toxoplasma gondii* infection among blood donors in Ardabil Province as main focus of zoonotic visceral leishmaniosis, northwestern Iran. Ann Parasitol. 2021;67(4):611-7.

174. Moshfe A, Arefkhah N, Sarkari B, Kazemi S, Mardani A. *Toxoplasma gondii* in blood donors: a study in boyer-ahmad county, Southwest Iran. Interdiscip Perspect Infect Dis. 2018;2018.

175. Barazesh A, Bolouki H, Obeidi N, Malekizadeh H, Shadvar N, Fouladvand MA. Evaluation of the Presence of Anti-*Toxoplasma* Antibodies in Blood Donors’ Presenting to Blood Transfusion Center, Bushehr, Iran. ISMJ. 2023;25(5):442-53.

176. Naeini KM, Soureshjani EH, Jafari M, Parchami S, Karimi G, Abdizadeh R. Prevalence of *Toxoplasma* *gondii* infection in healthy volunteer blood donors using serological and molecular methods from Chaharmahal and Bakhtiari Province, Southwest Iran. Jundishapur J Microbiol. 2019;12(5).

177. Ferdowsi S, Farsi L, Tajalli SM, Soltani H. Seroprevalence anti-*Toxoplasma* *gondii* antibodies and anti-epstein-barr virus (ebv) antibody among volunteer blood donors referred Gonabad Blood Transfusion Organization. J Rostamineh Zabol Univ Med. 2013.

178. Gholami m, Maghsood ah, Mohammadi a, Fallah n, Fallah m. Seroprevalence of Toxoplasmosis in blood donors of Hamadan transfusion center in 2013. YJMS. 2015;17(2):113-22.

179. Sadooghian S, Mahmoudvand H, Mohammadi MA, Sarcheshmeh NN, Kareshk AT, Kamiabi H, et al. Prevalence of *Toxoplasma* *gondii* Infection among healthy blood donors in Northeast of Iran. Iran J Parasitol. 2017;12(4):554.

180. Zainodini N, Mohammad Z-B, Abdollahi SH, Afrooz M, Ziaali N, Ebrahimian M, et al. Molecular and serological detection of acute and latent toxoplasmosis using real-time PCR and ELISA techniques in blood donors of Rafsanjan City, Iran, 2013. Iran J Parasitol. 2014;9(3):336.

181. Mahmoudvand H, Saedi Dezaki E, Soleimani S, Baneshi M, Kheirandish F, Ezatpour B, et al. Seroprevalence and risk factors of *Toxoplasma* *gondii* infection among healthy blood donors in south‐east of Iran. Parasite Immunol. 2015;37(7):362-7.

182. Saki J, Foroutan M, Khodkar I, Khodadadi A, Nazari L. Seroprevalence and molecular detection of *Toxoplasma gondii* in healthy blood donors in southwest Iran. Transfus Apher Sci. 2019;58(1):79-82.

183. Bahhaj R, Ahmadpour E, Mahami-Oskouei M, Fallah E, Shamsasenjan K, Safaiyan A. *Toxoplasma gondii* infection and related risk factors among blood donors in northwest Iran. Arch Clin Infect Dis. 2017;12(2).

184. Ormazdi H, Sanikhani N, Hadighi R, Akhlaghi L, Memar A, Razmju E. Investagation of Antibodies (IgG and IgM) against *Toxoplasma* *gondii* in Blood donor referred to Tehran Blood Transfusion Organization by ELISA. Stud Med Sci. 2010;21(2):212-6.

185. Shaddel M, Dizgah IM, Sharif F. The prevalence of toxoplasmosis in Imam Reza Hospital blood bank samples, Tehran, Iran. *Transfus Apher Sci*. 2014;51(2):181-3.

186. Kalantari N, Sheikhansari M, Ghaffari S, Alipour J, Gorgani-Firouzjaee T, Tamadoni A, et al. Seroprevalence and molecular detection of *Toxoplasma gondii* in young healthy blood donors in Northern Iran. Trop Biomed. 2018;35(4):1017-27.

187. Modrek MJ, Mousavi M, Saravani R. *Toxoplasma gondii* Seroprevalence Among Blood Donors in Zahedan, Southeastern Iran. Int J Infect. 2014.

188. Zarean M, Shafiei R, Gholami M, Fata A, Balaghaleh MR, Karimi A, et al. Seroprevalence of anti–*Toxoplasma* *gondii* antibodies in healthy voluntary blood Donors from Mashhad City, Iran. Arch Iran Med. 2017;20(7):441.

189. Ali-Asghari F, Shahri L, Besharati R, Arzamani K, Reaghi S. Frequency of IgG and IgM against *Toxoplasma gondii* in female students of North Khorasan University of Medical Sciences 2012-2013. JNKUMS. 2013;5:405-9.

190. Shafiei R, Shakeri HS. Seroprevalence of *Toxoplasma gondii* and Vitamin D3 Concentration among School Children 7-18 Years Old in Bojnurd City. J North Khorasan Univ Med Sci. 2023;14(4):30-7.

191. Asgari Q, Rajabi F, Sajadian F, Bahreini MS, Arefkhah N. *Toxoplasma gondii* infection in patients with brain tumors in Southern Iran: a case-control study. J Parasit Dis. 2023:1-6.

192. Arefkhah N, Asgari Q, Rajabi F, Sajadian F, Bahreini MS. Evidence of association of *Toxoplasma gondii* infection with brain tumors in Southern Iran: A case-control study. J Parasit Dis*.* 2022.

193. Kalantari N, Ghaffari S, Bayani M, Elmi MM, Moslemi D, Nikbakhsh N, et al. Preliminary study on association between toxoplasmosis and breast cancer in Iran. Asian Pac J Trop Biomed. 2015;5(1):44-7.

194. Nikbaksh N, Mazhari F, Bijani A, Rostami A, Bayani M. Protective effect of latent *Toxoplasma* infection against breast cancer risk: a comparative cross-sectional study in Iran. Curr Res Med Sci. 2022;6(1):34-8.

195. Mardani M, Tavalla M. Seroepidemiology of *Toxoplasma gondii* IgG and IgM among butchers in southwest of Iran. Asian Pac J Trop Dis. 2015;5(12):993-5.

196. Fouladvand M, Barazesh A, Naeimi B, Zandi K, Tajbakhsh S. Seroprevalence of toxoplasmosis in high school girls in Bushehr city South-west of Iran 2009. Afr J Microbiol Res. 2010;4(11):1117-21.

197. Fouladvand MA, Barazesh A, Naiemi B, Vahdat K, Tahmasebi R. Seroepidemiological Study of Toxoplasmosis in Girl Students from Persian Gulf University and Bushehr University of Medical Sciences. ISMJ. 2010;13(2):114-22.

198. Beheshtipour J, Adhami G, Moradi M, Shabani S. Seroprevalence and risk factors associated with toxoplasmosis among the butchers of Sanandaj City, west of Iran. Sci J Kurdistan Univ Med Sci. 2019;24(1):122-31.

199. Mahmoudi M, Rajabi E, Mirzaei A. *Toxoplasma gondii* infection in cancer patients in Guilan, Iran: prevalence and risk of acquired or reactivation of latent Toxoplasmosis. JBRMS. 2019;6(3):34-41.

200. Ghasemian M, Maraghi S, Saki J, Pedram M. Determination of antibodies (IgG, IgM) against *Toxoplasma gondii* in patients with cancer. Iran J Parasitol*.* 2007.

201. Getso MI, Raissi V, Fasihi Karami M, Alizadeh G, Zareie M, Babaei Samani Z, et al. Toxoplasmosis among patients with cancers and blood disorders in iran: Serological evaluation, risk factors and comparison with healthy individuals. J Chem Health Risks. 2021;11(3):271-82.

202. Khademvatan S, Khademvatani K, Tappeh KH, Asadi N, Khezri P, Abasi E. Association of *Toxoplasma* *gondii* infection with cardiovascular diseases: A cross-sectional study among patients with heart failure diseases in Urmia, North-West of Iran. Ann Parasitol. 2020;66(2).

203. Manouchehri Naeini K, Mortazaei S, Kheiry S. Seroepidemiology of Toxoplasma infection of women in child-bearing ages in Chaharmahal va Bakhtiyari province, Iran. Hormozgan Med J. 2014;17(6):505-14.

204. Sobati H. Chemiluminescent microparticle immunoassay-based detection and prevalence of *Toxoplasma gondii* infection in childbearing women (Iran). J Parasit Dis. 2020;44:273-80.

205. Raissi V, Bayat F, Taghipour A, Raiesi O, Ibrahim A, Getso M, et al. Seroepidemiology and risk factors of toxoplasmosis among children age ranged from 1 to 14 years referred to medical diagnostic laboratories in Southeast Iran. Clin Epidemiol Glob Health. 2020;8(2):595-99.

206. Rezavand B, Poornaki AM, Mokhtari KR, Mohammad A, Andalibian A, Abdi J. Identification and determination of the prevalence of *Toxoplasma gondii* in patients with chronic renal failure by ELISA and PCR. Asian Pac J Trop Dis. 2016;6(5):347-9.

207. Mardani A, Keshavarz H. Comparison 0f the two methods, IFA and ELISA, in seroepidemiological study of *Toxoplasma* infection in pregnant women of Qom City. J Sch Public Health Inst Public Health Res. 2004;2(3):57-64.

208. Fasihi-Karami M, Sayyah M, Kazemi F, Arjmand R. Comparison of Toxoplasmosis in Narcotic Drug-addicted and Healthy Persons in the Southwest of Iran; A Case-control Study. Cent Nerv Syst Agents Med Chem. 2023;23(1):65-70.

209. Afsharpaiman S, Khosravi MH, Faridchehr M, Komijani M, Radfar S, Amirsalari S, et al. Assessment of *Toxoplasma* seropositivity in children suffering from attention deficit hyperactivity disorder. Galen Med J. 2016;5(4):188-93.

210. Khademvatan S, Riahi F, Izadi-Mazidi M, Khajeddin N, Yousefi E. *Toxoplasma gondii* exposure and the risk of attention deficit hyperactivity disorder in children and adolescents. Pediatr Infect Dis J. 2018;37(11):1097-100.

211. Khodashenas S, Foroughi-Parvar F, Mosayebi M, Ghasemi M, Ghaleiha A, Tapak L. A case-control study of seroprevalence of *Toxoplasma* *gondii* in dementia patients in Arak and Hamadan, west of Iran. Arch Clin Infect Dis. 2019;14(5).

212. Nasirpour S, Kheirandish F, Fallahi S. Depression and *Toxoplasma gondii* infection: assess the possible relationship through a seromolecular case–control study. Arch Microbiol. 2020;202(10):2689-95.

213. Shamsinia S, Dalimi A, Pirestani M. Is Toxoplasmosis a risk factor in diabetic patients in Tehran? Infect Epidemiol Microbiol. 2019;5(3):49-59.

214. Khalili M, Mahami-Oskouei M, Shahbazi A, Safaiyan A, Mohammadzadeh-Gheshlaghi N, Mahami-Oskouei L. The correlation between serum levels of anti-*Toxoplasma gondii* antibodies and the risk of diabetes. Iran J Parasitol. 2018;13(4):637.

215. Shirbazou S, Delpisheh A, Mokhetari R, Tavakoli G. Serologic detection of anti *Toxoplasma gondii* infection in diabetic patients. Iran Red Crescent Med J. 2013;15(8):701.

216. Saki J, Shafieenia S, Foroutan-Rad M. Seroprevalence of toxoplasmosis in diabetic pregnant women in southwestern of Iran. J Parasit Dis. 2016;40:1586-9.

217. Shakiba N, Farhadifar F, Zareei M. Determine the IgG and IgM antibodies created against *T. gondii* infections using the ELISA method in diabetic pregnant women in compared with non-diabetic pregnant women in Sanandaj, Kurdistan, west of Iran. 2020. DOI: https://doi.org/10.21203/rs.3.rs-42507/v1

218. Khademvatan S, Khajeddin N, Saki J, Izadi-Mazidi S. Effect of toxoplasmosis on personality profiles of Iranian men and women. S Afr J Sci. 2013;109(1):1-4.

219. Saki J, Khademvatan S, Soltani S, Shahbazian H. Detection of toxoplasmosis in patients with end-stage renal disease by enzyme-linked immunosorbent assay and polymerase chain reaction methods. Parasitol Res. 2013;112:163-8.

220. Mansoori F. Seroepidemiology of toxoplasmosis in Kermanshah province, west of Iran, 2002. Behboud J Kermanshah Univ Med Sci. 2004;7(2):12-9.

221. Khatir AA, Moghaddam SA, Almukhtar M, Ghorbani H, Babazadeh A, Mehravar S, et al. *Toxoplasma* infection and risk of epilepsy: A case-control study of incident patients. Microb Pathog. 2021;161:105302.

222. Allahdin S, Khademvatan S, Rafiei A, Momen A, Rafiei R. Frequency of *Toxoplasma* and *Toxocara* sp. antibodies in epileptic patients, in south western Iran. Iran J Child Neurol. 2015;9(4):32.

223. Arefkhah N, Goodarzi R, Rezaei Z, Layegh Gigloo A, Sarkari B. Low prevalence of *Toxoplasma gondii* infection among children in a rural community in Fars province, southern Iran. Infez Med. 2019;27(3):322-27.

224. Hatam G, Shamseddin A, Nikouee F. Seroprevalence of Toxoplasmosis in High School Girls in Fasa District, Iran. Iran J Immunol. 2005;2(3).

225. Rahimi-Esboei B, Rahimi MT, Ghorbani A, Mahdavi SA. Genetic characterization of *Toxoplasma gondii* in women of reproductive age in the North of Iran. J Nurs Midwifery Sci. 2021;8(2):74.

226. Sharifi K, Sharifi K, Hoseini Farrash R, Khaledi A. Evaluate the sero-epidemiology of *Toxoplasma gondii* in patients referred to Ghaem hospital, Mashhad, Iran. Navid No. 2018;21(65):42-48.

227. Asmar M, Kh I, Masiha A. Prevalence of Serum Antibodies (IgM and IgG) *Toxoplasma gondii* in Guilan by ELISA Method. J Guilan Univ Med Sci. 2014;22(88).

228. Heidari A, Mokhtarian Daloie H, Ghahremani M, Basirat Moghaddam M, Moslem A, Sarshr N, et al. Seroepidemiological Study of Toxoplasmosis among High-School Girls in Gonabad. Qom Univ Med Sci J. 2011;5(3):57-63.

229. Rezayi Cherati F, Tohidi F, Fakhar M, Mohammadi R, Sharbatkhori M. Serological Evidence and Associated Risk Factors for *Toxoplasma* *gondii* in Gorgan and a Review of Recent Studies in Golestan Province. J Mazandaran Univ Med Sci. 2021;31(198):99-108.

230. Rabiee S, Fallah M, Shirmohammadi A, Serpoush H. Seroepidemiology of *Toxoplasma* infection in the women aged 15 to 45 years in Hamadan, West of Iran. J Res Health Sci. 2011;3(2):9-12.

231. Hamidi M, Khulojini M, Azizian R, Bashiri H, Ahanchian A, Babanejad M, et al. Seroprevalence of toxoplasmosis among women referring to Shahid Beheshti Hospital, Hamadan, Iran. Novelty Biomed. 2015;3(1):1-5.

232. Noorbakhsh S, Memari F, Farhadi M, Tabatabaei A. Sensorineural hearing loss due to *Toxoplasma gondii* in children: a case–control study. Clin Otolaryngol. 2008;33(3):269-73.

233. Hamidi F, Etemadi J, Ghabouli Mehrabani N, Mahami Oskouei M, Motavalli R, Ardalan MR. Comparison of *Toxoplasma gondii* seropositivity in hemodialysis and peritoneal dialysis patients. J Coast Life Med. 2015;3(3):621-22.

234. Kazemi F, Fallahizadeh S, Feizhadad MH. Seroepidemiological study of toxoplasmosis in hemodialysis patients of Ahvaz, Southwest of Iran. Int J Public Health. 2018;1(1):12-6.

235. Soltani S, Kahvaz MS, Soltani S, Maghsoudi F, Foroutan M. Seroprevalence and associated risk factors of *Toxoplasma gondii* infection in patients undergoing hemodialysis and healthy group. BMC Res Notes. 2020;13(1):1-5.

236. Seyyedpour SH, Afshar P, Barzegarnejad A, Kalhori S, Agah R. Evaluation of anti-*Toxoplasma gondii* antibodies in hemodialysis patients with chronic kidney disease in Sari, Iran. Nephrourol Mon. 2016;8(6).

237. Mirahmadi H, Mehravaran A, Sani Haidari M, Rahmati-Balaghaleh M, Raissi V, Shafiei R. Serological and molecular survey of *Toxoplasma gondii* infection in hemodialysis patients with chronic renal disease in Zahedan, Iran. J Kerman Univ Med Sci. 2021;28(4):391-98.

238. Zadeh AE, Bamedi T, Etemadi S, Shahrakipour M, Saryazdipour K. Toxoplasmosis as a complication of transfusion in hemodialysis patients. Iran J Ped Hematol Oncol. 2014;4(1):22.

239. Bayani M, Mostafazadeh A, Oliaee F, Kalantari N. The prevalence of *Toxoplasma gondii* in hemodialysis patients. Iran Red Crescent Med J. 2013;15(10).

240. Saadat F, Mahmoudi MR, Rajabi E, Roshan ZA, Shad BM, Karanis P. Seroepidemiology and associated risk factors of *Toxoplasma* *gondii* in hemodialysis patients. Acta Parasitol. 2020;65:906-12.

241. Fallahizadeh S, Feizhadad M-h, Kazemi F. Toxoplasmosis in hemodialysis and healthy individuals in Shush county, Southwest of Iran. Int J Public Health. 2018;1(3):122-26.

242. Alavi SM, Jamshidian R, Salmanzadeh S. Comparative study on *Toxoplasma* serology among HIV positive and HIV negative illicit drug users in Ahvaz, Iran. Caspian J Intern Med. 2013;4(4):781.

243. Abdollahi A, Shoar S, Sheikhbahaei S, Jafari S. Sero-prevalence of cytomegalovirus and *Toxoplasma* infections among newly diagnosed HIV patients in Iran, assessing the correlation with CD4+ cell counts. Iran J Pathol*.* 2013.

244. Daryani A, Sharif M, Meigouni M. Seroprevalence of IgG and IgM anti—*Toxoplasma* antibodies in HIV/AIDS patients, northern Iran. Asian Pac J Trop Med. 2011;4(4):271-4.

245. Nikbakht G, Behrouzi M, Mousavizadeh A, Pourabbas B, Rezaei Z, Nouripour-Sisakht S, et al. Seroprevalence of *Toxoplasma* *gondii* infection among HIV-positive patients in Southwest Iran and associated risk factors: a case-control study. Trans R Soc Trop Med Hyg. 2022;116(10):930-34.

246. Yazdani MR, Mehrabi Z, Ataei B, Ghahfarokhi AB, Moslemi R, Pourahmad M. Frequency of sero-positivity in household members of the patients with positive *Toxoplasma* serology. Rev Esp Quimioter. 2018;31(6):506.

247. Azami M. Seroprevalence of *Toxoplasma gondii* in women of child–bearing age referred to clinical laboratories in Ilam, western Iran. J Sci Technol Res. 2019;2(3)

248. Amirkhani A, Aghighi Z. Seroepidemiology and risk factors of toxoplasmosis in high school girls of Ilam in the year 2012. *J Adv Biomed* Sci. 2014;4(3):301-10.

249. Eskandarian A, Jahani S, Hejazi H, Yousefi H, Raissi V. Investigation of *Toxoplasma gondii* infection in Cutaneous Leishmaniasis patients of the Isfahan province. Int J Infect. 2017;4(2).

250. Mostafavi SN, Ataei B, Nokhodian Z, Yaran M, Babak A. Seroepidemiology of *Toxoplasma gondii* infection in Isfahan province, central Iran: A population based study. J Res Med Sci. 2011;16(4):496.

251. Mohaghegh MA, Yazdani H, Hadipour M, Namdar F, Azami M, Kalani H, et al. Seroprevalence of *Toxoplasma gondii* infection among patients admitted to Al-Zahra Hospital, Isfahan, Iran. *J Ayub Med Coll Abbottabad*. 2015;27(4):767-70.

252. Jafari R, Sadeghpour S, Sharifi F, Darani HY, Bagherpour B, Salar PS, et al. Anti-*Toxoplasma* IgM and IgG Seropositivity Among Individuals Referred to a Clinical Laboratory of Isfahan, Central Iran. Int J Infect. 2017;4(2).

253. Mostafavi N, Ataei B, Nokhodian Z, Monfared LJ, Yaran M, Ataie M, et al. *Toxoplasma gondii* infection in women of childbearing age of Isfahan, Iran: A population-based study. Adv Biomed Res. 2012;1.

254. Mahmoodi M, Mohebali M, Hejazi H, Keshavarz H, Alavi Naeini A, Izadi S. Seroepidemiological study on toxoplasmic infection among high-school girls by Ifat in Esfahan City, Iran. J Sch Public Health Inst Public Health Res. 2005;3(1):29-42.

255. Davami M, Pourahamd M, Sotoodeh Jahromi A, Tadayon S. *Toxoplasma* seroepidemiology in women who intend to marry in Jahrom, Islamic Republic of Iran. EMHJ. 2013;19.

256. Fallah Ismail MJ, Nawazesh Reza, Kushawar Hossein, Mehdipourzare Nasrin. . Epidemiology study of *Toxoplasma* infection in high school girls of Jolfa city. J Reprod Infertil. 2005;6(23):261-69.

257. Elyasi H, Souizi B. Seroepidemiology of toxoplasmosis and its risk factors in newly married women of Jovin city (Razavi Khorasan) in 1400. J Zoonoses Dis. 2022;2(2):30-40.

258. Parvizpour F, Hajighasemlo S, Hasani S, Olfati L, Bahmani A, Hoseini F, et al. Toxoplasmosis infection in the pregnant women in the first half of pregnancy, in Kamyaran in 2008. Sci J Kurdistan Univ Med Sci. 2010;15(1):72-8.

259. Arbabi M, Farzadfar HS, Houshyar H. Prevalence of *Toxoplasma* *Gondii* infection in single women referring to Kashan health centers (2007-2008). Daneshvar Med. 2009;16(4):7-12.

260. Hooshyar H, Bagherian T, Heidarzadeh Z, Baghbani F. Seroprevalence Of Toxoplasmosis in Women Referred To Kashan Reference Laboratory 2008-2012. J Toloo Behdasht. 2015;14(1).

261. Rasti S, Hooshyar H, Arbabi M, Fatahian A, Behrashi M, Talebian A, et al. Frequency of *Toxoplasma* infection among pregnant women and their newborn in Kashan, Iran. Zahedan J Res Med Sci. 2015;17(6).

262. Keshavarz H, Mamishi S, Daneshvar H. The prevalence of *Toxoplasma* infection in hospitalized patients in selected hospitals of Kerman. J Kerman Univ Med Sci. 2000;7(3):129-36.

263. Majid HGHAARAbMHMpHSFFAREH. Serum Prevalence of Anti-*Toxoplasma* IgG Antibody Among Female Students of Kerman University of Medical Sciences, Academic Year 2013-2014. Iran J Infect Dis Trop Med. 2008;13(41):39-43.

264. Narooi Dehnavi M, Nourollahi Fard SR, Khovand H, Sakhaee E. Seroepidemiologic study on the prevalence of anti-*Toxoplasma* *gondii* antibodies in referred women to south of Kerman Province laboratories. JJUMS. 2018;5(1):259-66.

265. Tavakoli Kareshk A, Keyhani A, Asadi A, Zia-Ali N, Mahmoudvand H, Mohammadi AR. Seroprevalence of *Toxoplasma gondii* infection among childbearing age women in Kerman city, southeastern Iran. J Parasit Dis. 2016;40:1544-47.

266. Pouyanmehr M, Mirbahari S, Ghaffarzadeh S. Study of Sero-prevalence of IgM and IgG Antibodies to *Toxoplasma* Infection. Iran J Allergy Asthma Immunol. 2018;17.

267. Pooyanmehr M, Nik Ghalb A, Darabi M. Seroprevalence of toxoplasmosis in Kermanshah, A cross-sectional study. NFVM. 2021;3(2):45-55.

268. Kalani H, Mohammadi F, Faridnia R, Mirzaei F, Virgilio S, Heydarian P, et al. Seroprevalence of toxoplasmosis in Kermanshah City, west of Iran. Ann Parasitol. 2021;67(2):229-36.

269. Almasian R, Almasian M, Zibaei M. Sero-Epidemiology of toxoplasmosis among the people of Khorram Abad. J Infect Dis Ther. 2014;2(5):1-3.

270. E B. Prevalence of *Toxoplasma gondii* in pregnant women referring to Khorramabad health centers. YJMS*.* 3. 2001;9:33-35.

271. Yad MJY, Jomehzadeh N, Sameri MJ, Noorshahi N. Seroprevalence of anti-*Toxoplasma* *gondii* antibodies among pregnant woman in South Khuzestan, Iran. Jundishapur J Microbiol. 2014;7(5).

272. Mamishie S, Syadatie A, Keshavarz H, Holakoui K. Prevalence of Toxoplasmosis in Patients Admitted to Outpatient Department in Markaze Tebi Koudakan. J Guilan Univ Med Sci. 2003;12(46):93-104.

273. Mehrabani D, Motazedian MH, Asgari Q, Mehrabani G. Sero-prevalence of *Toxoplasma* *gondii* in two migrating Lore and Khamseh tribes in southern Iran. Galen Med J. 2014;3(4).

274. Khayat NM, Ghareh DY. Survey OF *Toxoplasma* contamination in malignant canceric patients by ELISA method and comparison it with control group in Tabriz IN TBRIZ (1386). J Vet Microbiol. 2009.

275. Nasimi A, Fani M, Salehi M, Ghasemi H, Nezami H, Haghighi FH. Seroprevalence of *Toxoplasma gondii*, *Rubella* and *Cytomegalovirus* Among Women of Reproductive Age in Mashhad, Northeast of Iran. Europe PMC*.* 2021. https://doi.org/10.21203/rs.3.rs-1184788/v1.

276. Hosseini SA, Golchin E, Sharif M, Sarvi S, Ahmadpour E, Rostamian A, et al. A serological investigation and genotyping of *Toxoplasma* *gondii* among Iranian blood donors indicates threat to health of blood recipients. Transfus Apher Sci. 2020;59(3):102723.

277. Sharif M, Daryani A, Ebrahimnejad Z, Gholami S, Ahmadpour E, Borhani S, et al. Seroprevalence of anti-*Toxoplasma* IgG and IgM among individuals who were referred to medical laboratories in Mazandaran province, northern Iran. J Infect Public Health. 2016;9(1):75-80.

278. Khalili B, Javanmardi F, Mortazaei S, Ghasemi-Dehkordi P. Seroepidemiological Study of *Toxoplasma* *gondii* Infection of Mentally Retarded Patients (Chahrmahal Va Bakhtiari Province, Iran). *J Pure Appl Microbiol*. 2014;8(3):2193-97.

279. Soltan Mohammad Zadeh M, Keshavarz H, Mohebali M, Holakouie Naieni K, Arshi S. Seroepidemiologic study of human *Toxoplasma* infection in residents of Meshkin-Shahr. J Sch Public Health Inst Public Health Res. 2003;1(4):57-72.

280. Yaghoub F, Yagoob G. Seroprevalence of *Toxoplasma gondii* infection in pregnant women in Miandoab city, Iran. JLS. 2014;4(3):449-54.

281. Kamal DJRSAIKRJ. Serologic prevalence of human toxoplasmosis in the Mianeh city By Chemiluminescence's method. JLACSR. 2011;5(1):33-42.

282. J Davoodi MS, A Bahman Shabestari , S Rasouli ,A Khodadadi , K Jafary. Survey on serologic prevalence of human toxoplasmosis in males and females referred to Centeral Medical Laboratory in the Mianeh city by Elisa method. J Vet Clin Pathol. 2012;6(1 (21) ):1435-45.

283. Jouyani N, Ahady MT, Abbasi V. Serum level of anti-*Toxoplasma gondii* IgG among the individuals with/without migraine. JSSU. 2020.

284. Bitaraf HR, Arab-Mazar Z, Ghanimatdan M, Mohammadi M, Mohseni M, Parsipour S, et al. Seroprevalence of *Toxoplasma gondii* in military personnel and their families referred to the military hospital in Tehran, Iran. Novelty Biomed. 2017;5(4):152-7.

285. Kheirandish F, Nazari H, Mahmoudvand H, Yaseri Y, Tarahi MJ, Fallahi S, et al. Possible link between *Toxoplasma gondii* infection and mood disorders in Lorestan province, Western Iran. Clin Infect Dis. 2016;11.

286. Choobdarian H, Khadem Erfan MB, Zamini G, Foroutan P, Faridi A, Javan K, et al. Comparison of serum level of *Toxoplasma gondii* antibody between patients with multiple sclerosis and healthy people. Int J Biomed Public Health. 2019;2(4):66-68.

287. Keighobadi M, Alokandeh ND, Baghbanian SM, Karimi N. The role of *Toxoplasma gondii* in multiple sclerosis: A matched case-control study. Neurol Asia. 2021;26(2).
