# Supplementary material for: Anti-Toxoplasma gondii IgG seroprevalence in the general population in Iran: A systematic review and meta-analysis, 2000–2023
Source: PLoS One. 2024 Aug 29;19(8):e0307941. doi: 10.1371/journal.pone.0307941 (PMC11361691; doi:10.1371/journal.pone.0307941)
Supplement: Attachment — Submitted filename: Reviewer comments.docx [file pone.0307941.s002.docx]

The manuscript " *Anti-Toxoplasma gondii IgG seroprevalence in the general population in Iran: A systematic review and meta-analysis, 2000–2023*" presented the anti-*T. gondii* IgG seroprevalence within the general population in Iran, a large country in the Middle East. These article kinds are significant for increasing the sensitivity of the medical community and health policymakers especially in developing countries.

The article structure is acceptable, but the authors should answer the questions and make changes based on the comments.

Abstract

- The abstract should not be presented in a structured format
- *" Chronic toxoplasmosis is associated with several neuropsychiatric and other harmful effects in infected people, emphasizing the need to assess its burden across various world regions.”* → It is incorrect to mention the facts in the proof and hypothesis stage as absolutely proven.

Keywords

- Keywords must be in MeSH (Medical Subject Headings) term.
- Keywords must start with a capital letter
- Keywords must be given in alphabetical order.

Introduction

-	Using appropriate and detailed references in all parts of the manuscript. In a review article, referring to other review articles must be done with reason and logic, otherwise, it is unacceptable.

- References 2 to 5 should be removed, because they are unrelated to text and will lead to self-citation, which is against publication ethics.
- “*Recent studies have proposed links between chronic toxoplasmosis infection and psychiatric disorders like schizophrenia [10], depression [9], bipolar disorder [10], and epilepsy [11], underscoring the importance of reliable diagnosis of chronic toxoplasmosis within the general population.*“ → Summarize and use original articles for reference.
- “*The exposition of T. gondii in the Iranian population has been mostly approached by the detection of anti-Toxoplasma IgG by serological methods like enzyme-linked immunosorbent assay (ELISA), and indirect immunofluorescence antibody test (IFA)”* → Applied methods in Iran have not been fully addressed
- *“While several meta- analysis studies focused on T. gondii occurrence in immunocompromised individuals such as cancer patients [6, 13-15]”* → There is no need to mention other meta-analyses and different study groups. Be omitted.
- *"IGP"* In the last sentence of the introduction → Be corrected

Methods

- *“The search terms used encompassed "Toxoplasma gondii", "T. gondii", "toxoplasmosis", "Iran", "Islamic Republic of Iran", "seroprevalence", and "general population".”* → It is necessary to mention the strategic syntax search
- *“general population”* is not MeSH term
- A number of keywords are missed: ‘*Toxoplasma*”, “seropositivity” and “prevalence”

Results

- Considering in the analysis table, the results of the cities of Mazandaran, Khuzestan, Qom, Kurdistan, Gilan, Kermanshah, Qazvin, Lorestan, Yazd, Fars, Hormozgan, Semnan, Kerman, and Sistan and Baluchistan have more different results compared to Kalantari *et al.* that recently published study and you just mention that in the introduction. Are the results of this study not included in the above analysis? What argument would you make on this?

Discussion

- Reference 22 is not relevant and needs to be removed
- Reference 33 is not relevant and needs to be removed
- “*Also, in this study, the highest seroprevalence was observed among pregnant women. Considering that the risk of contracting this parasite during pregnancy is clinically important [45], we suggest that preventive measures to prevent pregnant women from becoming infected with T. gondii should be strictly applied. However, it should be noted that infection before pregnancy causes resistance to infection and transmission to the fetus. As a result, IgM identification is important in pregnant women [45].*” → Use original articles for reference, also, considering the main topic of the article, it is not relevant to deal with pregnant women.

First, the "Disclosures" section should be mentioned, and then "Compliance with Ethics Guidelines" should be mentioned.

References

- The second reference must be correctly given. This book is not published in 2020.
